# Gelatin Hydrogel Crosslinking: From Molecular Design to Functional Soft Materials

**DOI:** 10.3390/gels12090798

**Published:** 2026-09-02

**Authors:** Pietro Tordi

**Affiliations:** Department of Chemistry “Ugo Schiff” and CSGI, University of Florence, via della Lastruccia 3, 50019 Sesto Fiorentino, Florence, Italy; pietro.tordi@unifi.it

**Keywords:** gelatin, hydrogel, crosslinking, drug release, tissue engineering, water remediation, food packaging, sensing, energy storage

## Abstract

Gelatin hydrogels combine biological origin, biodegradability, abundant chemical functionality, and broad processability, but their native thermoreversible physical networks generally lack the stability required for sustained operation. Crosslinking overcomes this limitation while regulating hydration, mechanics, transport, degradation, and responsiveness. This review examines gelatin hydrogel crosslinking from a structure–property–function perspective, connecting molecular design with physicochemical characterization and functional performance. Temperature-induced gelation and ion-mediated physical interactions are compared with small-molecule- and coupling-agent-mediated, enzyme-catalyzed, and photoinduced covalent crosslinking strategies, highlighting their different balances among reversibility, stability, processability, and biocompatibility. Particular attention is given to the characterization methods required to relate junction chemistry and network organization to swelling, thermal behavior, mechanical response, degradation, and molecular or ionic transport. These relationships are evaluated across drug delivery and controlled release, tissue engineering and wound healing, food packaging, preservation and delivery, water remediation and environmental management, wearable sensing and bioelectronics, and energy storage. Across these fields, the central challenge is not to maximize crosslinking, but to balance network stability with the molecular mobility required for function. By integrating complementary crosslinking mechanisms with multiscale characterization, gelatin can be engineered as a programmable platform for advanced soft materials.

## 1. Introduction

Biopolymer hydrogels are hydrated networks formed from naturally derived macromolecules that can retain large amounts of water while preserving a defined three-dimensional structure. Their softness, permeability, high water content, biodegradability, and chemical functionality make them attractive sustainable soft materials across biomedical, environmental, food-related, and energy-oriented technologies [1,2,3,4,5,6]. Their performance depends not only on the selected biopolymer, but also on network architecture, water–polymer interactions, mechanical stability, and transport properties. Therefore, biopolymer hydrogel design requires rational control over the interactions that connect macromolecular building blocks into stable and functional networks [7,8,9,10].

Among natural polymers, gelatin holds a prominent position because it combines biological origin, broad availability, low cost, processability, biodegradability, and chemical versatility. Gelatin is obtained by partial hydrolysis of collagen and retains many molecular features that make collagen-derived materials attractive for soft-matter and biointerface applications. Depending on the extraction route, gelatin is commonly classified as type A or type B, obtained under acidic or alkaline conditions, respectively [11,12,13]. These processing conditions influence the isoelectric point, charge distribution, molecular weight, amino acid composition, and chain-length distribution of the resulting material. Commercial gelatin is also commonly described by its Bloom strength, which reflects gel firmness and is related to molecular weight distribution, chain organization, and physical network formation. These feedstock characteristics also influence crosslinking by affecting chain organization, reactive-site accessibility, and the ability of gelatin to reform triple-helical junctions. Consequently, gelatin source, extraction history, molecular-weight distribution, and Bloom strength can contribute substantially to differences in crosslinking efficiency and network properties among studies.

Native gelatin hydrogels form through reversible triple-helical junctions upon cooling, enabling mild aqueous processing but making network integrity strongly dependent on temperature, pH, ionic strength, hydration, and thermal history [11,12,13]. This physical reversibility can lead to softening, swelling, dissolution, mechanical instability, and rapid degradation under application-relevant conditions. Crosslinking is therefore required not merely to stabilize gelatin, but to control the permanence and dynamics of the network while retaining the hydration and processability of the native material. By introducing additional non-covalent or covalent junctions, crosslinking enables the coordinated tuning of mechanical integrity, swelling, molecular and ionic transport, degradation, shape retention, and responsiveness, although increased stabilization may also restrict mobility and degradability.

Because gelatin hydrogels are used in very different environments, no single crosslinking strategy is universally optimal. Temperature-induced gelation provides the native physical network, offering mild processing and thermoreversibility but limited long-term stability. Ion-mediated interactions can further regulate gelatin organization, either by modifying chain hydration and charge screening or by reinforcing hybrid networks containing ionically active polymers [14,15]. Small-molecule crosslinkers and coupling agents typically introduce covalent junctions that improve stability and resistance to dissolution, although their use must be balanced against cytotoxicity, network heterogeneity, and reduced degradability [16,17,18]. Enzymatic crosslinking offers a milder alternative, forming selective covalent bonds under aqueous and biologically compatible conditions [19], while photocrosslinking provides temporal and spatial control through modified gelatin derivatives such as GelMA and related photoresponsive systems [20]. Thus, the choice of crosslinking route should be guided by the target function rather than by crosslinking density alone.

Crosslinking has consequently enabled the use of gelatin in controlled release, tissue regeneration, food-related materials, environmental remediation, soft bioelectronics, and energy storage [21,22,23,24,25,26]. Across these fields, it acts not merely as a stabilization step, but as a molecular design tool governing hydration, transport, deformation, degradation, and responsiveness.

Previous reviews have addressed gelatin from a broad materials perspective [12] or focused on specific application areas, including tissue engineering [22], food packaging [23], wearable electronics [25], and energy storage [26], as well as individual crosslinking chemistries such as genipin [27] and gelatin methacryloyl (GelMA) [28]. These contributions provide substantial insight within their respective scopes, but the relationships among crosslinking mechanism, network structure, physicochemical properties, and functional performance across different fields remain fragmented. The present review addresses this gap by comparatively examining temperature-induced gelation, ion-mediated interactions, small-molecule and coupling-agent crosslinking, enzymatic crosslinking, and photocrosslinking within a common structure–property–function framework. This analysis is extended across six major application domains, spanning drug delivery, tissue engineering and wound healing, food-related materials, environmental remediation, wearable sensing and bioelectronics, and energy storage. By integrating crosslinking chemistry, network organization, physicochemical properties, and application-specific performance within a single framework, the review provides a unified perspective that is not limited to a single crosslinking route or application field.

## 2. Crosslinking Strategies

Crosslinking defines the stability, organization, and functional behavior of gelatin hydrogels by regulating the nature, density, and reversibility of the junctions connecting the protein chains. For clarity, the term crosslinking is used here in a broad network-design sense, while the individual mechanisms are distinguished according to the nature of the junctions formed. Temperature-induced gelatin gelation is treated as reversible physical gelation; ion-induced changes in gelatin itself are described as physical association, reinforcement, or network reorganization; ionic crosslinking is reserved for gelatin-containing hybrid networks in which multivalent ions form junctions primarily through an ionically active secondary polymer; and chemical, enzymatic, and photoinduced reactions that generate persistent covalent junctions are classified as covalent crosslinking. The following sections examine temperature-induced gelation and ion-mediated interactions alongside small-molecule- and coupling-agent-mediated, enzyme-catalyzed, and photoinduced covalent routes. Each strategy offers a distinct balance among processing simplicity, mechanical reinforcement, transport, degradability, and biocompatibility. Their principal molecular features, advantages, and limitations are comparatively summarized at the end of Section 2.

### 2.1. Physical Crosslinking

#### 2.1.1. Temperature-Induced Gelation

Temperature-induced gelation is the native and most fundamental form of physical crosslinking in gelatin hydrogels. Unlike covalent crosslinking, it relies on reversible non-covalent associations between gelatin chains. Although the partial hydrolysis and denaturation of collagen disrupt its native fibrillar architecture, gelatin retains glycine-, proline-, and hydroxyproline-rich sequences capable of partial helix reformation. Above the sol–gel transition temperature, the chains are predominantly present as hydrated random coils, whereas cooling promotes their reorganization into collagen-like triple-helical domains. These ordered regions act as physical junction zones connecting flexible disordered chains within a water-swollen three-dimensional network. Hydrogen bonding, hydrophobic interactions, van der Waals forces, and chain entanglement further contribute to network cohesion, accounting for the softness, high water content, and thermoreversibility of native gelatin gels, but also for their limited mechanical and thermal stability [12,29,30,31].

The final properties of these physical networks are governed by both gelatin characteristics and processing history. Polymer concentration, molecular weight distribution, Bloom strength, source, extraction route, and type A/type B classification determine chain overlap, charge distribution, hydration, and the availability of helix-forming segments. At the same time, cooling rate, gelation temperature, aging time, and thermal cycling control the nucleation, growth, and maturation of triple-helical junctions. Together, these factors regulate junction-zone density, chain packing, swelling, and gel strength. Higher gelatin concentration and Bloom strength, together with slower cooling and longer aging, generally favor more cohesive networks, although the final response remains sensitive to pH, ionic strength, and hydration history.

This thermal sensitivity facilitates processing but limits long-term use. Gelatin can be molded, thermally reshaped, or injected as a warm solution before gelation under mild aqueous conditions, which is advantageous for food-related materials, biomedical formulations, and temporary soft matrices. However, the same reversible junctions can be disrupted by heating, dilution, changes in pH or ionic strength, and prolonged immersion in water. Since the gel–sol transition often occurs close to biologically or technologically relevant temperatures, purely physical gelatin gels may lose shape, dissolve, swell excessively, or undergo structural aging during use.

#### 2.1.2. Ion-Mediated Interactions and Reinforcement

Ion-mediated interactions offer a useful route for regulating gelatin hydrogel structure, although their role differs from conventional ionic crosslinking in polyanionic polysaccharides. Unlike alginate, where multivalent cations can form the primary network [32,33], gelatin remains mainly dependent on the temperature-driven reformation of collagen-like triple-helical domains. Ions therefore modulate this physical network rather than replace it, making ion-mediated reinforcement or network reorganization more appropriate descriptions than classical ionic gelation.

Gelatin ion sensitivity arises from its amphoteric character and heterogeneous charge distribution. Far from the isoelectric point, the greater magnitude of the net chain charge promotes electrostatic repulsion, chain expansion, and hydration, whereas reduced repulsion near the isoelectric point facilitates closer packing and intermolecular association [11,12,13]. The different isoelectric-point ranges of type A and type B gelatin can consequently lead to distinct responses under the same ionic conditions. Added ions further alter this balance through charge screening, changes in protein hydration, transient coordination with protein residues, and, in some cases, ion-mediated associations between chains. Because these processes are coupled to the coil-to-helix transition, their net effect may be reinforcing or destabilizing.

The influence of cations cannot be predicted from valence alone. Depending on ion identity and concentration, monovalent and divalent cations have been reported to decrease modulus and melting temperature or delay gelation by interfering with the formation of triple-helical junctions. Conversely, strongly coordinating multivalent ions, such as Fe^3+^, may densify and stiffen the network through additional ion–protein associations. These contacts are more heterogeneous and less structurally defined than alginate-type egg-box junctions, and their contribution depends on ion concentration, hydration, size, polarizability, coordination affinity, pH, and thermal history [14,34]. In this context, experimentally measured changes in modulus, melting temperature, gelation kinetics, and nanoscale network organization demonstrate that metal ions alter gelatin network behavior, but they do not by themselves establish direct ion–gelatin coordination. Unless coordination is supported by independent molecular evidence, these effects should therefore be interpreted as potentially arising from a combination of specific ion–protein interactions, electrostatic screening, and changes in hydration.

**Figure 1:**
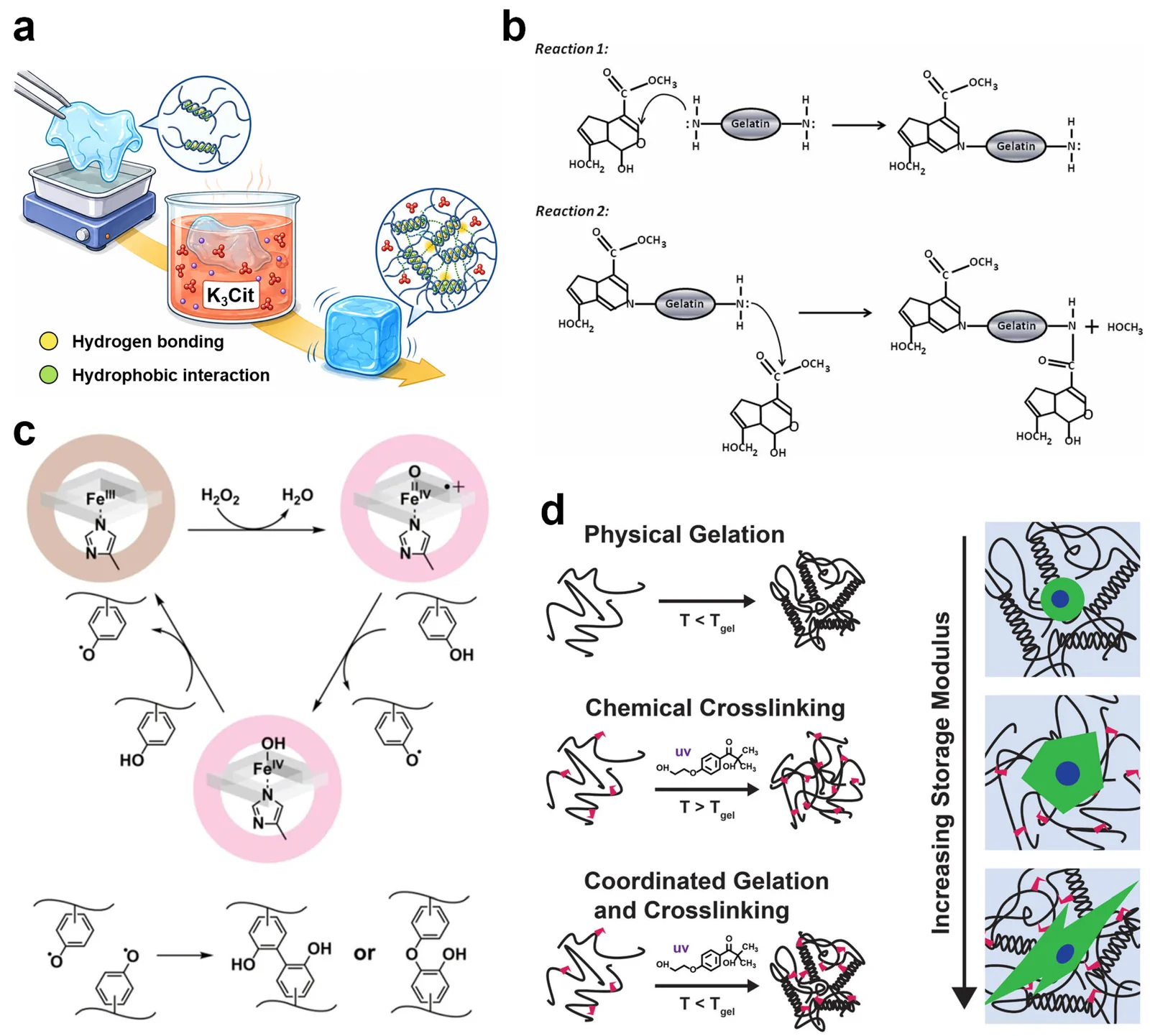
(**a**) Schematic representation of Hofmeister-mediated reinforcement induced by soaking gelatin in K_3_Cit solution, promoting protein dehydration, triple-helix formation and packing, hydrogen bonding, and hydrophobic association. Inspired by the work of Wang et al. [36]. (**b**) Proposed genipin crosslinking mechanism involving reactions with gelatin primary amino groups and subsequent covalent intermolecular coupling. Reproduced with permission from [16]. (**c**) Horseradish peroxidase (HRP)/H_2_O_2_ catalytic cycle involving oxidation of phenolic groups to phenoxy radicals, followed by radical coupling and crosslinking of phenol-functionalized polymer chains. Reproduced with permission from [42]. (**d**) GelMA network formation through temperature-induced physical gelation below the gelation temperature (T < T_gel_), UV-induced photocrosslinking in the presence of Irgacure 2959 above the gelation temperature (T > T_gel_), or their combined action below T_gel_. The resulting differences in network organization and storage modulus influence adherent-cell morphology. Magenta symbols indicate methacryloyl groups, while green and blue represent cells and nuclei, respectively. Reproduced with permission from [43].

Anions can exert similarly important effects through Hofmeister-type interactions. Kosmotropic anions such as sulfate, phosphate, and citrate may promote protein dehydration and intermolecular association, facilitating triple-helix formation and strengthening the physical network (Figure 1a). By contrast, more chaotropic anions, particularly thiocyanate, can disturb water-mediated stabilization or interact with the peptide backbone, thereby hindering the coil-to-helix transition. These effects remain strongly dependent on concentration and environmental conditions [35,36]. Hofmeister ordering should therefore be regarded as a qualitative tendency rather than a universal predictor of gelatin reinforcement, because specific ion effects can vary with concentration, pH, gelatin type, temperature, and the composition of hybrid networks.

The role of ions becomes particularly important in hybrid hydrogels, where gelatin is combined with biopolymers possessing stronger ionic or coordinating functionality. This complementary distribution of roles is well illustrated by alginate–gelatin networks, in which multivalent ions predominantly coordinate alginate carboxylates to establish the ionic scaffold, while gelatin contributes softness, elasticity, thermoresponsiveness, and water retention [37]. A similar interplay occurs in caseinate–gelatin systems, where metal ions organize the caseinate-rich phase and gelatin provides a compliant protein matrix, with both components contributing to hydration and mechanical behavior [38]. Related ion-regulated behavior can occur in gelatin hybrids containing carrageenan, carboxymethyl cellulose, or chitosan, although the dominant interactions depend on the charge and coordination chemistry of the secondary polymer [39,40,41]. The division of roles is nevertheless not absolute, because the ionic environment can also modify gelatin hydration and triple-helical organization, thereby affecting swelling, transport, mechanics, and network stability. Gelatin is therefore better regarded as an ion-sensitive and mechanically active component than as either a passive filler or a conventional ionic gelator. Representative examples of the ion-mediated, covalent, enzymatic, and photoinduced network-forming mechanisms discussed in the following sections are summarized in Figure 1a–d.

### 2.2. Chemical Crosslinking

#### 2.2.1. Covalent Crosslinkers and Coupling Agents

Covalent crosslinkers and coupling agents stabilize gelatin either by becoming incorporated into network junctions or by promoting bond formation between reactive groups already present along the protein chains. Reactions involving primarily amino and carboxyl groups supplement the native thermoreversible associations with more persistent junctions, improving thermal stability and resistance to dissolution. The resulting swelling, degradation, and mechanical properties depend on crosslinker structure and concentration, reaction conditions, diffusion through the network, and post-crosslinking purification. Excessive crosslinking or residual reagents may nevertheless compromise biocompatibility, degradability, and molecular transport. This coexistence of native physical and introduced junctions is an important feature of chemically stabilized gelatin networks. In a single gelatin network, stabilization may be dominated by one type of junction, whereas dual-crosslinked systems combine physical and covalent or dynamic junctions within the same polymer network. Interpenetrating networks instead contain two independently crosslinked polymer networks that are physically interlaced. In gelatin, the balance between these architectures is strongly influenced by thermal history, reaction sequence, and the relative kinetics of physical and chemical gelation, because covalent stabilization may either preserve preformed triple-helical domains or restrict their subsequent formation.

Aldehyde-based reactions represent the classical approach to covalent gelatin stabilization. Glutaraldehyde reacts primarily with lysine-derived amino groups, initially forming imine bonds that can evolve into more complex and stable covalent structures [44,45,46,47]. This chemistry efficiently increases stiffness and water stability but is limited by residual reactivity, potential cytotoxicity, and the formation of heterogeneous networks when crosslinker diffusion and reaction rates are poorly balanced. Its use in biomedical systems therefore requires careful control of reagent concentration, reaction time, and post-crosslinking purification.

Oxidized dextran, oxidized alginate, dialdehyde starch, and related polysaccharides provide macromolecular alternatives to conventional aldehyde crosslinkers [48,49,50]. Their aldehyde groups react with gelatin amino groups through Schiff-base chemistry, simultaneously forming covalent junctions and introducing a second biopolymer component. Depending on aldehyde content and network formulation, the resulting imine bonds may retain partial reversibility under aqueous conditions, enabling injectable, self-healing, or pH-responsive behavior. These systems therefore extend aldehyde chemistry from permanent stabilization toward dynamic covalent networks, although their long-term stability remains sensitive to hydrolysis and environmental conditions.

Genipin offers a naturally derived alternative to highly reactive synthetic crosslinkers. It reacts mainly with primary amino groups through a complex sequence of reactions that generates stable covalent junctions and the characteristic blue coloration of genipin-crosslinked protein networks (Figure 1b) [16,27,51,52,53,54]. Compared with glutaraldehyde, genipin is generally associated with lower cytotoxicity, although its biocompatibility remains dependent on crosslinker concentration, reaction extent, residual unreacted genipin, and the biological model and exposure conditions used for evaluation. Its reaction also proceeds more slowly and remains strongly influenced by pH, temperature, and formulation. Genipin can substantially improve mechanical integrity and resistance to dissolution while generally retaining enzymatic degradability, although at a reduced rate, making it attractive when network stability must be balanced against biological compatibility.

Carbodiimide-mediated coupling provides a mechanistically different route. The most widely used system combines 1-ethyl-3-(3-dimethylaminopropyl)carbodiimide with N-hydroxysuccinimide to activate carboxyl groups and promote amide-bond formation with gelatin amino groups [55,56,57]. This process is described as zero-length crosslinking because the coupling agent does not remain as a spacer within the final network [58,59]. It therefore stabilizes gelatin while minimizing the introduction of foreign structural elements. Its efficiency depends strongly on pH, reagent stoichiometry, functional-group accessibility, hydrolysis of the activated intermediates, and competition between intermolecular and intramolecular coupling. High degrees of crosslinking may also restrict swelling and molecular transport or reduce the accessibility of biologically active sequences. These chemistries therefore involve distinct trade-offs in reaction kinetics, aqueous processability, residual reactivity, purification burden, bond stability, reversibility, and degradation. Because these parameters are strongly formulation- and protocol-dependent and scalability is rarely evaluated on a common basis, direct cross-study ranking is generally not meaningful; crosslinker selection should instead be guided by application-specific requirements.

#### 2.2.2. Enzymatic Crosslinking

Enzymatic crosslinking offers an attractive route for stabilizing gelatin hydrogels under mild aqueous conditions. Unlike conventional small-molecule crosslinkers, enzymes catalyze selective activation or coupling reactions involving specific amino acid residues or chemically introduced functionalities. Covalently stabilized networks can therefore be generated under comparatively mild, often near-physiological conditions while limiting the use of highly reactive crosslinking reagents. These characteristics are particularly advantageous for biomedical, food-related, and cell-laden systems in which cytocompatibility and controlled gelation are essential.

Among the available enzymatic systems, microbial transglutaminase (mTG) is the most extensively investigated for gelatin stabilization. It catalyzes an acyl-transfer reaction between the γ-carboxamide groups of glutamine residues and the ε-amino groups of lysine residues, generating stable ε-(γ-glutamyl)lysine isopeptide bonds [60,61,62,63]. Because both residues are naturally present in gelatin, mTG can directly connect protein chains without requiring prior chemical functionalization. The resulting covalent networks generally exhibit enhanced mechanical integrity, thermal stability, and resistance to dissolution compared with physically gelled gelatin. The established use of mTG in food processing also makes this approach particularly relevant to edible hydrogels and food-compatible soft materials.

The properties of mTG-crosslinked gelatin depend on enzyme concentration, reaction time, temperature, and the organization and accessibility of the protein chains during crosslinking. This aspect is particularly important because gelatin simultaneously undergoes temperature-dependent physical assembly through the partial reformation of collagen-like triple-helical domains. The final network architecture therefore reflects the interplay among enzyme kinetics, thermal history, physical gelation, and substrate accessibility rather than enzyme concentration alone.

Horseradish peroxidase (HRP) provides another widely investigated route and is generally combined with hydrogen peroxide to crosslink gelatin derivatives bearing phenolic groups, such as tyramine- or hydroxyphenyl-modified gelatin (Figure 1c) [42,64,65,66]. HRP catalyzes the oxidation of phenolic groups to phenoxy radicals, which subsequently undergo coupling to generate covalent junctions between neighboring chains. Variation in the enzyme and hydrogen peroxide concentrations enables control over gelation kinetics and network density, while rapid reaction under physiological conditions is advantageous for injectable hydrogels, cell encapsulation, and biofabrication. Nevertheless, the degree of phenolic functionalization must be carefully controlled because extensive modification may alter the biological characteristics and degradability of gelatin. Excessive hydrogen peroxide concentrations may also induce oxidative damage or reduce cell viability.

Tyrosinase and laccase further extend oxidative enzymatic crosslinking [67,68]. Tyrosinase catalyzes the hydroxylation of monophenolic groups and the oxidation of catechols to reactive quinones, which can subsequently react with amines, thiols, or other nucleophiles. This mechanism is particularly relevant to adhesive catechol-modified gelatin hydrogels. Laccase promotes the one-electron oxidation of phenolic groups using molecular oxygen as the terminal oxidant, generating radical intermediates that undergo subsequent coupling without requiring hydrogen peroxide. Both approaches often require or benefit from prior gelatin functionalization and careful control of oxidation kinetics.

Enzymatic crosslinking should therefore distinguish direct coupling of native gelatin residues, as in mTG-mediated reactions, from routes that rely on prior chemical functionalization, as in many HRP-based systems. The latter introduce an additional modification step that may contribute to batch variability and affect degradability or biological functionality independently of the subsequent enzymatic reaction.

Overall, enzymatic crosslinking combines mild processing with chemical selectivity, but its performance remains dependent on substrate accessibility, enzyme activity, reaction kinetics, and, for oxidative systems, the controlled generation of reactive intermediates.

#### 2.2.3. Photocrosslinking

Photocrosslinking enables gelatin hydrogel networks to be formed with high spatial and temporal control. Unlike thermally induced physical gelation or covalent reactions initiated directly by reagent mixing, light exposure can trigger network formation on demand, allowing rapid crosslinking and spatial patterning. This combination of controllable processing and the biological properties of gelatin has made photocrosslinking particularly valuable for biofabrication, injectable hydrogels, cell-laden constructs, and functional soft materials.

The most widely investigated photocrosslinkable gelatin derivative is gelatin methacryloyl (GelMA). It is commonly prepared by reacting gelatin with methacrylic anhydride, which introduces polymerizable methacryloyl functionalities primarily at lysine residues (Figure 1d) [28,43,69,70]. Upon irradiation in the presence of a photoinitiator, the introduced carbon–carbon double bonds undergo radical polymerization, generating a covalently stabilized network. GelMA retains many features of the parent protein, including cell-interactive motifs and enzymatically degradable sequences, while introducing a light-triggered mechanism for network formation. It has therefore become a widely used platform for hydrogel biofabrication.

GelMA properties are governed by the degree of methacryloyl substitution, gelatin concentration, and photopolymerization conditions [71]. Increasing the number of reactive groups generally produces denser networks with higher stiffness, lower swelling, slower degradation, and reduced molecular transport. Excessive modification, however, may restrict chain mobility and partially compromise the biological functionality of gelatin, whereas insufficient functionalization may result in weak or poorly stable networks. The degree of substitution must therefore be selected according to the required balance among mechanical stability, transport, degradability, and biological performance. Accordingly, GelMA should not be regarded as a chemically uniform material, and meaningful comparison among studies requires reporting of the degree of methacryloylation together with the relevant preparation and photocrosslinking conditions.

Photoinitiator chemistry and irradiation conditions provide further control over network formation. Wavelength, light intensity, exposure time, initiator concentration, oxygen availability, and sample thickness influence radical generation, conversion, and crosslinking homogeneity. Near-UV irradiation with initiators such as Irgacure 2959 was historically widespread, but limited penetration and potential damage to cells or biomolecules have encouraged the adoption of violet- and visible-light systems [72,73]. Lithium phenyl-2,4,6-trimethylbenzoylphosphinate (LAP) enables rapid polymerization under near-UV or violet light [72,74], whereas eosin Y-based formulations permit visible-light crosslinking in the presence of suitable coinitiators [75,76]. These systems should not be compared on irradiation wavelength alone, because initiator chemistry, concentration, photoproducts, radical-generation efficiency, and required exposure conditions all contribute to the biological response. Consequently, shifting from UV to violet or visible light does not automatically ensure cytocompatibility, which remains dependent on both photochemical composition and total irradiation dose. Photocrosslinking must consequently be considered a coupled process in which polymer functionalization, initiator selection, irradiation conditions, and biological constraints are optimized together.

A mechanistically distinct visible-light strategy relies on ruthenium/sodium persulfate (Ru/SPS)-mediated photo-oxidative crosslinking. Unlike GelMA photopolymerization, this approach can directly stabilize unmodified gelatin by promoting covalent dityrosine formation between native tyrosine residues. Upon irradiation, the Ru(II) photosensitizer and persulfate generate oxidizing species that induce tyrosyl-radical formation and subsequent intermolecular coupling. This route therefore avoids prior methacryloylation or other introduction of polymerizable groups, although its efficiency depends on tyrosine availability, Ru/SPS concentration, light dose, and chain organization. Recent studies have demonstrated visible-light stabilization of native gelatin through this mechanism, with particularly effective network formation when photo-oxidative crosslinking is combined with prior temperature-induced physical gelation [77,78].

Light-triggered network formation is especially useful in additive manufacturing and microfabrication because it controls shape fidelity, printing resolution, and structural stability. Rapid crosslinking limits construct deformation, whereas excessive irradiation or network densification may produce brittle structures, restrict molecular diffusion, and reduce cell viability. Spatially controlled illumination can also generate stiffness gradients, microchannels, anisotropic regions, and locally defined transport properties. Photocrosslinking therefore serves not only as a stabilization method, but also as a tool for programming hydrogel architecture.

Alternative gelatin derivatives have been developed to overcome some limitations of conventional methacrylate polymerization. Norbornene-functionalized gelatin undergoes thiol–ene photopolymerization with multifunctional thiols [79,80,81]. These reactions proceed through a step-growth mechanism that generally produces more homogeneous networks, lower sensitivity to oxygen inhibition, and greater control over crosslinking architecture than conventional chain-growth polymerization. This modular chemistry facilitates the preparation of interpenetrating, composite, or multifunctional networks in which gelatin provides hydration and biological functionality while secondary components regulate mechanical and responsive properties.

Despite these advantages, many widely used photocrosslinking strategies require prior chemical modification and purification of gelatin, potentially introducing batch-to-batch variability. Photoinitiators and radical intermediates may cause cytotoxicity, oxidative stress, or damage to encapsulated biomolecules, particularly at high concentrations or prolonged exposure times. Light attenuation in thick, opaque, or strongly absorbing samples can also produce gradients in conversion and mechanical properties. Oxygen inhibition, incomplete polymerization, local heating, and residual unreacted groups represent additional sources of variability.

The final network is also influenced by gelatin’s native thermoreversible assembly. Photocrosslinking above the gelation temperature can lock the chains in a predominantly disordered state, whereas irradiation after cooling stabilizes a preorganized network containing triple-helical domains. These processing pathways may produce different swelling, mechanical, degradation, and transport properties even at comparable degrees of functionalization. Gelatin photocrosslinking should therefore be understood as an interplay between photoinduced covalent network formation and temperature-dependent physical assembly. Table 1 provides a comparative overview of the network-forming strategies discussed in Section 2.

## 3. Physicochemical Characterization of Gelatin Hydrogels

A reliable interpretation of crosslinked gelatin hydrogels requires complementary characterization across multiple length and time scales. Chemical analyses establish whether the intended reaction or functionalization has occurred, whereas microscopy and scattering reveal the resulting network organization and heterogeneity. Hydration, thermal, rheological, and mechanical measurements then connect this structure to stability, transport, deformation, and degradation. The following sections therefore illustrate how these techniques can be integrated to establish meaningful relationships between crosslinking chemistry, network architecture, and macroscopic performance; representative quantitative examples of these structure–property relationships are summarized at the end of Section 3. A recurring methodological limitation is that gelatin concentration, crosslinker dose, and degree of functionalization are often varied simultaneously, so changes in mechanical, swelling, or degradation behavior cannot be attributed to crosslink density alone. These parameters should therefore be treated as independent formulation variables when establishing structure–property relationships.

Crosslinking density is rarely measured directly in gelatin hydrogels. Gel fraction reports the insoluble network fraction, while free-amine assays and degree of substitution quantify reactive-group consumption or functionalization rather than intermolecular junction density. Swelling and equilibrium or rheological moduli provide indirect estimates that depend on assumptions about network homogeneity and polymer–solvent interactions, whereas spectroscopic changes mainly support chemical conversion. These proxies are therefore not interchangeable and should be interpreted together rather than as absolute measures of crosslinking density [28,82,83,84,85,86]. Accordingly, each characterization method is considered not only in terms of the information it provides, but also in terms of the interpretations that it cannot support independently.

### 3.1. Chemical Confirmation of Crosslinking

Chemical characterization is essential for interpreting crosslinked gelatin hydrogels because the formation of a self-supporting gel does not necessarily demonstrate that the intended crosslinking reaction has occurred. Gelatin can form physical networks through temperature-induced triple-helix formation, while changes in pH, ionic strength, solvent composition, drying, or polymer blending may also alter consistency and stability without generating new covalent junctions. The chemical nature and extent of the reaction should therefore be assessed before changes in swelling, degradation, mechanical performance, or biological response are attributed to crosslinking.

Fourier-transform infrared spectroscopy is among the most widely used techniques for this purpose. In gelatin hydrogels, changes in the amide regions can provide information on peptide-group interactions, hydrogen bonding, and secondary-structure variations [86,87,88,89,90,91]. In crosslinked systems, FTIR can also reveal shifts or intensity changes associated with specific reactions. However, such spectral changes are not, by themselves, definitive evidence of covalent bond formation and should be supported by appropriate controls or complementary chemical analyses. Aldehyde-based crosslinking may be accompanied by signals related to imine formation and reduced availability of primary amines, whereas carbodiimide-mediated coupling can affect carboxylate and amide-related bands. In functionalized systems, such as gelatin methacryloyl, FTIR can support the introduction of polymerizable groups, although it should preferably be combined with more quantitative methods [92,93,94].

Nuclear magnetic resonance spectroscopy is particularly useful when gelatin is chemically modified before hydrogel formation. In GelMA, proton NMR is commonly used to identify methacrylate or methacrylamide signals and to estimate the degree of functionalization [94,95]. This quantification is important because the density of reactive functionalities directly influences gelation kinetics, crosslinking density, swelling, degradation, stiffness, and biological performance.

Colorimetric assays provide a simple route to quantify the consumption of reactive amino groups. Ninhydrin and TNBS assays are commonly used to estimate free primary amines before and after crosslinking or functionalization [96,97,98,99,100]. They are especially relevant for reactions involving lysine residues, including glutaraldehyde, genipin, transglutaminase, and methacryloylation. A decrease in free amino groups supports successful reaction, but it should not be interpreted alone as proof of an effective network, because amino-group consumption may arise from grafting, intramolecular reactions, or incomplete intermolecular coupling.

Complementary techniques should be selected according to the crosslinking chemistry. X-ray photoelectron spectroscopy is useful for films, coatings and hybrid hydrogels because it provides information on surface composition and chemical environments [101,102]. Because XPS probes only the near-surface region and is usually performed on dried samples, it may not reflect the bulk hydrated network. Crosslinking should therefore be confirmed by combining chemical analysis with measurements of gel fraction, sol fraction or resistance to dissolution.

### 3.2. Physical Network Structure and Morphology

Once the crosslinking chemistry has been assessed, structural characterization is needed to determine how the gelatin network is organized across different length scales. Gelatin hydrogels are intrinsically heterogeneous and their properties depend on chain packing, triple-helical domains, crosslinking junctions, water-rich regions and pore architecture. In hybrid systems they also depend on the distribution of secondary polymers, particles or inorganic phases. Crosslinking may densify the network, alter chain organization, suppress dissolution or generate local heterogeneity. Structural and morphological analyses therefore connect molecular crosslinking with swelling, degradation, transport and mechanical performance.

**Figure 2:**
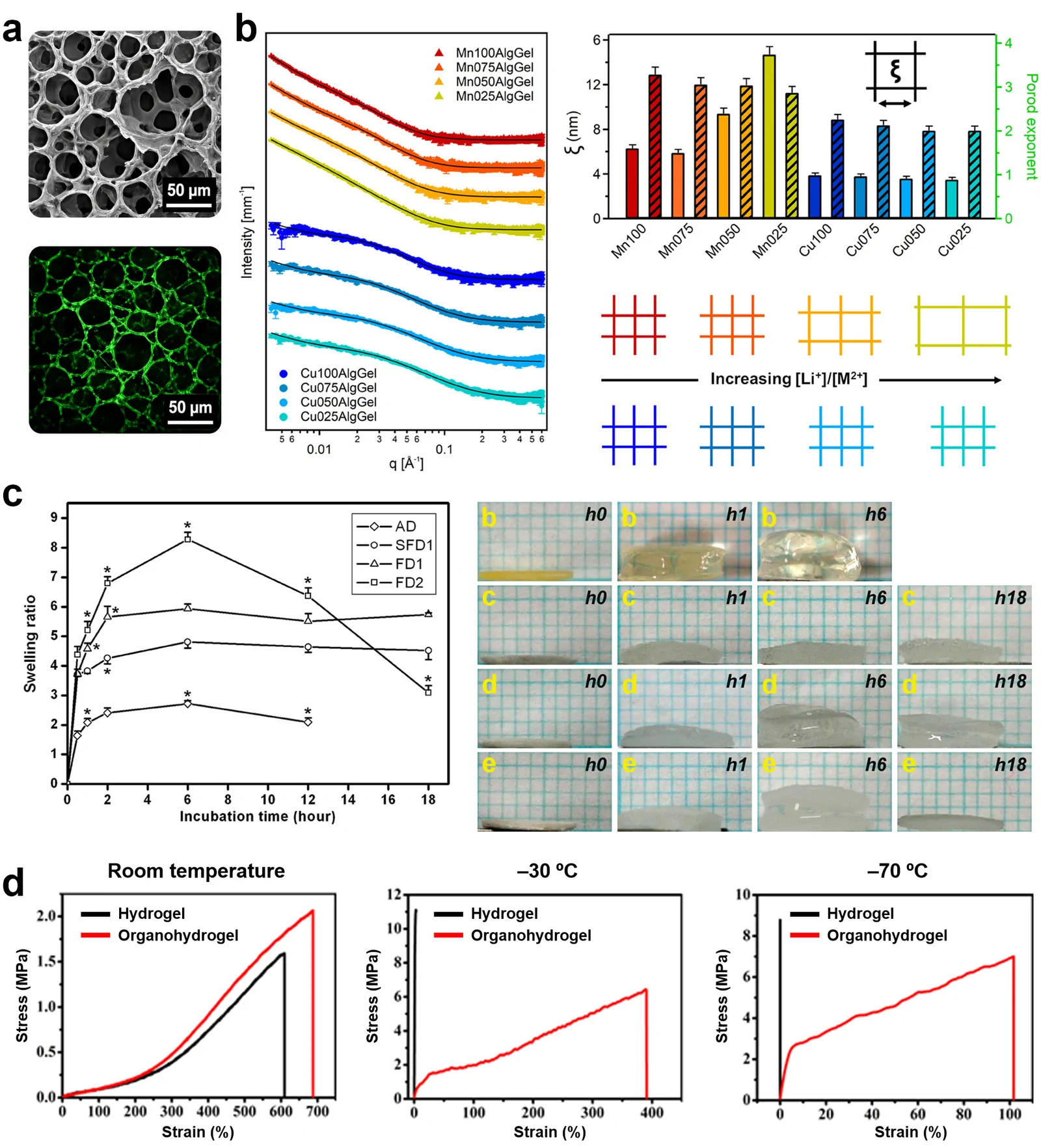
(**a**) Conceptual illustrations of porous hydrogel morphologies visualized by scanning electron microscopy (top) and fluorescence microscopy (bottom). (**b**) SAXS profiles, correlation lengths (*ξ*), and Porod exponents of MnXAlgGel and CuXAlgGel networks, where Mn^2+^ or Cu^2+^ acts as the crosslinking ion and X denotes the Li^+^/M^2+^ ratio. Increasing Li^+^ content loosens MnXAlgGel networks, whereas CuXAlgGel remains comparatively compact. Reproduced with permission from [121]. (**c**) Swelling of EDC-crosslinked gelatin discs in balanced salt solution at 34 °C. AD, SFD1, FD1, and FD2 denote air-dried, stirred/frozen at −20 °C, frozen at −20 °C, and frozen at −196 °C samples, respectively. Photographs show the samples before and after incubation; each grid square corresponds to 1 mm. Asterisks indicate significant differences from the preceding time point (*p* < 0.05; *n* = 4). Adapted with permission from [122]. (**d**) Tensile stress–strain curves of citrate-treated gelatin hydrogels and corresponding water/glycerol organohydrogels (1:1, *w*/*w*) at room temperature, −30 °C, and −70 °C. Adapted with permission from [123].

Scanning electron microscopy is widely used to examine the porous morphology of gelatin hydrogels. After freeze-drying or critical-point drying, SEM can reveal pore dimensions, connectivity, wall thickness, surface roughness and large-scale differences between formulations (Figure 2a) [86,103]. Highly crosslinked networks often appear denser and less porous, whereas weakly stabilized gels may display larger and more open pores. However, SEM images must be interpreted carefully because drying can strongly alter the original hydrated structure. In many cases, the observed morphology reflects the freeze-dried scaffold rather than the true network architecture in water.

Atomic force microscopy provides complementary information on surface organization and local heterogeneity. In gelatin films, coatings, or thin hydrogels, AFM can reveal nanoscale roughness, fibrillar features, phase separation, and local stiffness variations [104,105,106,107]. AFM nanoindentation is especially useful for cell-contacting materials because cells respond to local mechanical cues rather than only to bulk modulus. In crosslinked gelatin hydrogels, this technique can show whether the network is mechanically uniform or whether crosslinking generates domains with different stiffness, adhesion, or surface organization.

Confocal laser scanning microscopy (Figure 2a) is particularly useful for hydrated and three-dimensional samples. When gelatin hydrogels are fluorescently labeled, loaded with particles, or combined with cells, confocal microscopy can provide spatial information on internal architecture, filler distribution, cell localization, and degradation patterns [108,109,110,111]. This is important for tissue engineering, drug delivery, and biofabrication, where performance depends on cell infiltration, nutrient transport, and molecular diffusion. In printed or patterned gelatin hydrogels, confocal microscopy can also be used to evaluate layer organization, shape fidelity, and spatial distribution of functional components.

Small-angle X-ray scattering (Figure 2b) and small-angle neutron scattering provide access to smaller structural length scales than microscopy. Their main advantage is that hydrated hydrogels can be analyzed closer to their native state, without complete drying. In gelatin systems, SAXS and SANS can provide information on nanoscale heterogeneity, correlation length, chain organization, aggregation, and ordered domains associated with triple-helix formation [112,113,114,115,116]. These techniques are therefore valuable for connecting crosslinking chemistry with mesoscale network organization. Two hydrogels may appear similar by SEM but differ substantially in hydrated chain packing, junction distribution, or nanoscale density fluctuations.

X-ray diffraction can be useful when hydrogels contain ordered domains, crystalline fillers, mineral phases, or inorganic components [117,118,119,120]. Native gelatin usually displays broad features associated with its largely amorphous protein structure, but changes in triple-helical ordering or filler crystallinity can be detected in specific systems. For this reason, XRD is less generally informative for simple gelatin hydrogels than microscopy or scattering, but it becomes important for mineralized scaffolds, composite gels, and hybrid materials containing crystalline phases.

### 3.3. Hydration and Thermal Properties

Hydration and thermal behavior are central to gelatin hydrogels because gelatin is both water-rich and thermoresponsive. Water acts not only as the solvent retained inside the network, but also as a structural component that controls chain mobility, swelling, diffusion, softness, and degradation. At the same time, gelatin can reorganize with temperature through helix formation and melting. Crosslinking therefore affects hydrogel performance by changing both how much water the network absorbs and how strongly this water is associated with the polymer chains.

Swelling experiments are among the simplest methods for evaluating hydrogel hydration (Figure 2c) [122,124,125]. The equilibrium swelling ratio indicates how much water the network can absorb, whereas time-dependent profiles describe the kinetics of water uptake. These profiles can be fitted using empirical models such as the Weibull equation to extract characteristic time and shape parameters, enabling quantitative comparison among different formulations [126]. In general, denser crosslinked networks swell less because chain expansion is more restricted. However, swelling should not be interpreted as a direct measure of crosslinking density alone, since it also depends on pore structure, hydrophilicity, charge state, degradation, and chain packing. For this reason, swelling data are most meaningful when interpreted together with chemical and morphological characterization.

Differential scanning calorimetry provides information on both thermal transitions and water organization. In gelatin hydrogels, DSC can be used to study helix–coil transitions, melting of ordered domains, and changes in thermal behavior induced by crosslinking. Chemical or enzymatic crosslinks may shift, broaden, or suppress these transitions by restricting chain mobility and stabilizing the network [127,128,129]. DSC can also distinguish freezable and non-freezable water [130,131,132]. Freezable water behaves more similarly to bulk water and contributes to crystallization or melting peaks, whereas non-freezable water is more strongly associated with polymer chains and does not crystallize under conventional cooling conditions. This distinction is useful because bound water strongly influences softness, transport, and low-temperature stability.

Thermogravimetric analysis complements DSC by quantifying water loss, residual solvent, and thermal degradation [130,133,134]. The first mass-loss step is generally associated with evaporation of water or other volatile components, while higher-temperature events correspond to degradation of gelatin and any crosslinkers, plasticizers, salts, or secondary polymers. Crosslinked gelatin hydrogels often show improved thermal stability compared with native gelatin, although the magnitude of this effect depends on the chemistry and density of the junctions. TGA is especially useful for comparing dried or partially dried hydrogels, films, aerogels, cryogels, and composite systems.

Thermal characterization should also account for processing history. Cooling rate, aging time, storage temperature, hydration state, and repeated heating–cooling cycles can affect triple-helix formation, physical junction density, and network stability. Even when gelatin is chemically crosslinked, residual physical junctions may persist and reorganize with time or temperature. Therefore, thermal analysis is not only a stability assessment, but also a way to understand how physical and chemical crosslinks coexist within the same material.

### 3.4. Mechanical and Functional Characterization

Mechanical and functional characterization determines whether crosslinking provides gelatin hydrogels with the stability and performance required for their intended use. Because wound dressings, scaffolds, food matrices, printable bioinks, sensors, and energy-storage devices operate under different conditions, hydrogel performance cannot be adequately described by a single modulus value. Instead, the selected measurements should reflect the deformation modes, operating environment, and functional requirements of the target application.

Rheology is particularly useful for investigating gelatin gelation and viscoelastic behavior. Time sweeps can follow the evolution of the storage and loss moduli during network formation, whereas frequency and strain sweeps provide information on network strength, relaxation behavior, linear viscoelastic range, and resistance to structural breakdown [135,136,137]. These measurements are especially informative when physical gelation and covalent crosslinking occur simultaneously or on overlapping timescales, as in enzymatically or photochemically stabilized gelatin systems. A single storage-modulus value should therefore not be interpreted as a direct measure of crosslinking density, particularly in heterogeneous or physically associated gelatin networks. Frequency dependence and the presence of an apparent plateau region, together with relaxation behavior and time-dependent gelation kinetics, provide a more informative description of junction persistence and structural evolution.

Bulk mechanical tests provide complementary information on resistance to deformation and failure. Compression testing is commonly used for soft hydrated materials, scaffolds, and food gels [138,139], whereas tensile testing is more relevant to films, wearable devices, and highly deformable hydrogels (Figure 2d) [123,140]. Young’s modulus, compressive strength, elongation at break, toughness, and fracture energy describe different aspects of mechanical behavior and should be selected according to the application. Although greater network stabilization often enhances stiffness and strength within a given formulation, this trend is not universal because changes in crosslinker concentration can simultaneously modify network organization, hydration, chain mobility, and secondary interactions. Excessive crosslinking may also reduce extensibility, increase brittleness, and limit energy dissipation.

Dynamic measurements are required when hydrogels experience sustained or repeated loading. Cyclic compression or tension, stress-relaxation, creep, and recovery tests can reveal hysteresis, fatigue resistance, viscoelastic dissipation, and structural recovery. These properties may be more representative of practical performance than initial stiffness, particularly for injectable materials, wearable sensors, and other systems subjected to repeated deformation.

Stability and degradation measurements evaluate whether the crosslinked network remains intact under relevant environmental conditions [141,142,143]. Gelatin hydrogels may undergo dissolution, thermal softening, hydrolysis, or enzymatic degradation, and crosslinking can regulate the rate of these processes. Tests performed in water, buffers, enzymatic media, or application-specific environments should therefore monitor mass loss, dimensional stability, swelling, and retention of mechanical properties over time.

Functional characterization depends more directly on the intended use. Drug-delivery, wound-healing, and active-packaging systems require release, diffusion, and permeability measurements to determine how network density, hydration, degradation, and polymer–solute interactions regulate molecular transport. For sensing and energy-storage applications, ionic conductivity, impedance, capacitance, gauge factor, response time, hysteresis, and cycling stability become relevant. In these systems, crosslinking must balance mechanical durability with ion mobility and water retention. Functional measurements should therefore be interpreted together with structural, hydration, and mechanical data and, whenever possible, performed under realistic deformation, temperature, humidity, and cycling conditions. Table 2 provides representative quantitative examples of the structure–property relationships discussed in Section 3.

## 4. Applications

Across the following application sections, the discussion is organized around a common structure–property–function framework, considering how the selected crosslinking strategy modifies network architecture and physicochemical properties and how these changes translate into functional performance. Particular attention is given to application-specific trade-offs and to cases in which comparable changes in network stabilization produce different outcomes because of coupled formulation and processing variables.

These six application areas were selected because they impose distinct and complementary network requirements, allowing the same crosslinking principles to be examined under different demands on stability, transport, degradation, mechanical response, and ionic or molecular mobility.

The functional versatility of gelatin hydrogels arises from the possibility of adapting crosslinking chemistry and network architecture to markedly different operating environments. Drug delivery and tissue-engineering systems require cytocompatibility, controlled degradation and molecular transport. Food packaging, preservation and delivery demand moisture control, barrier performance and safe formulations, whereas water remediation and environmental management require aqueous stability and accessible functional sites. Wearable sensing, bioelectronics and energy storage instead depend on specific combinations of deformability, conductivity, ionic mobility and operational stability. The following sections examine how these requirements are addressed across the six application fields. The corresponding network requirements, crosslinking objectives, performance indicators and principal trade-offs are summarized at the end of Section 4.

### 4.1. Drug Delivery and Controlled Release

Drug delivery is among the most established biomedical applications of gelatin-based hydrogels because gelatin combines high water affinity, biodegradability, enzymatic susceptibility and abundant chemical functionality with the ability to accommodate therapeutic molecules within a hydrated polymer network. Native gelatin is rarely sufficient as a delivery matrix because it swells rapidly, has limited stability under physiological conditions and often releases encapsulated molecules too quickly. Crosslinking is therefore commonly required not only to preserve hydrogel structure but also to regulate mesh size, swelling, degradation, drug–matrix interactions and the balance between burst and sustained release. Release profiles from gelatin-based networks can also be interpreted using kinetic models such as zero-order, first-order, Higuchi, and Korsmeyer–Peppas descriptions. The Higuchi model is commonly used to assess diffusion-dominated release from matrix systems, whereas the Korsmeyer–Peppas exponent can help distinguish predominantly Fickian transport from anomalous behavior involving coupled diffusion and polymer relaxation. Crosslinking can therefore modify not only the release rate but also the apparent transport regime by altering mesh size, swelling, chain mobility, and degradation [148,149]. Depending on the formulation, release may be dominated by diffusion through the hydrated network, swelling-induced changes in mesh size, network degradation, or affinity interactions between the therapeutic molecule and the matrix. Crosslinking can shift the relative contribution of these mechanisms by modifying mesh size and tortuosity, swelling kinetics, degradation rate, and the accessibility of drug-binding sites.

This structure–release relationship was already evident in early gelatin delivery systems. Glutaraldehyde-crosslinked gelatin hydrogels were used for the local dual release of cisplatin and adriamycin, enabling trans-tissue chemotherapy with distinct release profiles for the two drugs [150]. Related strategies have been applied to antibiotic delivery, including gelatin microspheres for vancomycin and jellyfish gelatin hydrogels for cefazolin release. Increasing the degree of glutaraldehyde crosslinking generally restricts swelling and reduces the effective network mesh size, thereby slowing drug diffusion, whereas lower crosslinking promotes faster release [151]. The study of Charoenchokpanich et al. on jellyfish gelatin further showed that gelatin source and chain architecture influence gel fraction, swelling and antibiotic release. Release behavior therefore depends not only on the crosslinker but also on the intrinsic properties of the gelatin precursor [152].

Genipin provides a less cytotoxic alternative for tuning gelatin-based delivery systems. Genipin-crosslinked gelatin matrices showed that crosslinking temperature can act as a simple processing parameter to regulate network structure. Higher temperatures produced denser microstructures, reduced swelling, increased puncture strength and slowed indomethacin release while preserving cytocompatibility [145]. Genipin has also been incorporated into chitosan–gelatin ocular hydrogels, where co-crosslinking contributed to sustained timolol maleate release and improved ocular retention compared with conventional eye-drop administration [153]. These examples show that release can be adjusted without modifying the drug itself by changing the crosslinking chemistry, crosslinking degree or processing conditions.

Injectable and in situ-forming hydrogels represent another important direction, particularly when the carrier must conform to irregular defects or sensitive anatomical sites. Enzymatically crosslinked gelatin–phenol hydrogels based on HRP/H_2_O_2_ provide rapid gelation under mild conditions, allowing the drug depot to form directly after injection [91]. The incorporation of oxidized β-cyclodextrin into gelatin–tyramine networks further demonstrates how crosslinking can be combined with host–guest interactions. Schiff-base bonds improved adhesion and resistance to degradation, while cyclodextrin cavities enhanced the solubilization and release of hydrophobic drugs such as dexamethasone and curcumin [154]. In a related enzymatic approach, transglutaminase-crosslinked gelatin–alginate coatings were used to deliver gentamicin or vancomycin from titanium implants. Increasing the transglutaminase content produced more strongly stabilized coatings and extended antibiotic release from minutes to several days, reducing infection-related tissue damage in vivo, particularly in the vancomycin-loaded group [155].

Photocrosslinked GelMA hydrogels add spatial and temporal control over network formation. In diabetic wound healing, deferoxamine (DFO)-loaded GelMA acted as both a scaffold and a therapeutic reservoir, releasing DFO rapidly enough to stimulate early angiogenic signaling. In vitro experiments showed fibroblast adhesion and HIF-1α/VEGF-related responses, whereas in vivo studies demonstrated vascularization, granulation tissue formation, and re-epithelialization [156]. In ocular delivery, injectable GelMA hydrogels loaded with triamcinolone acetonide showed that GelMA concentration controls pore size, swelling, degradation and release duration. Denser networks reduced swelling, slowed degradation and extended release compared with free drug suspensions while maintaining retinal compatibility in vivo, illustrating how network density can regulate therapeutic retention without compromising biological performance [157].

Taken together, crosslinking in gelatin-based delivery systems should be designed to match network stability and degradation with the required release timescale rather than simply minimize molecular diffusion. Excessive network densification may improve retention but can also restrict swelling, degradation, and therapeutic release, making the optimal crosslinking conditions strongly dependent on the drug, administration route, and intended treatment duration.

### 4.2. Tissue Engineering and Wound Healing

Tissue engineering is one of the most established biomedical applications of crosslinked gelatin hydrogels because gelatin combines a collagen-derived composition with high hydration, biodegradability and intrinsic cell-interactive motifs. These features allow gelatin-based networks to reproduce several aspects of the extracellular matrix and support cell adhesion, proliferation, migration and remodeling. Native gelatin is generally unsuitable for long-term regenerative applications because physically formed gels are thermoreversible, mechanically weak and unstable under physiological conditions. Crosslinking is therefore generally required to preserve scaffold shape, regulate water uptake, resist premature dissolution and match degradation with tissue formation. The degree of crosslinking must remain carefully balanced because excessive stabilization may restrict swelling, diffusion and cell invasion, whereas insufficient crosslinking may lead to rapid collapse or loss of scaffold integrity [63,158].

A major application concerns cutaneous, oral and soft-tissue repair. Gelatin hydrogels can act as hydrated wound matrices, bioactive dressings or temporary scaffolds that maintain a moist environment while supporting cellular infiltration and tissue remodeling. Gelatin hydrogel sheets containing concentrated platelet lysate enhanced wound healing in mice, particularly at two- and three-fold lysate concentrations, leading to improved granulation tissue formation, capillary development and epithelialization [159]. Chitosan–gelatin hydrogels have also been optimized as wound-dressing matrices, showing that polymer composition and crosslinker content strongly influence swelling, porosity and degradation [160]. Genipin-crosslinked gelatin–hyaluronic acid patches have been proposed for inflammatory skin conditions such as atopic dermatitis, where crosslinking improved flexibility, hydration-related properties and structural stability compared with non-crosslinked gels [161].

Oral and gingival regeneration further illustrates how crosslinking chemistry governs scaffold performance at the cell–material interface. Glutaraldehyde-crosslinked gelatin hydrogels have been used to examine how crosslinking degree modifies surface properties and human gingival fibroblast attachment, showing that cell response is highly sensitive to network density and surface mechanics [162]. EDC/NHS-crosslinked gelatin hydrogels provide a zero-length coupling route based on direct amide-bond formation without introducing an additional molecular spacer into the network. In gingival fibroblast studies, these hydrogels showed improved pore interconnectivity, greater viscoelastic stability, reduced dissolution at 37 °C, preserved cell viability, enhanced migration and increased COL1 expression, supporting their potential for gingival tissue regeneration at the in vitro level [163].

Enzymatic crosslinking is particularly relevant when viable cells must be encapsulated or when gelation must occur under mild conditions. Microbial transglutaminase-crosslinked gelatin hydrogels supported HEK293 cell proliferation within the matrix and allowed recolonization of culture surfaces after proteolytic release. These systems remained stable at 37 °C, while degradation could be tuned through gelatin concentration and the combined contribution of physical gelation and enzymatic crosslinking [63]. In myocardial tissue engineering, direct comparison of mTG-, genipin-, glutaraldehyde- and EDC-crosslinked gelatin showed that the mTG-crosslinked network produced the most favorable cardiomyocyte response. It supported adhesion, survival, spontaneous beating, maturation-marker expression, calcium transients and electrical propagation, whereas the genipin-, glutaraldehyde- and EDC-crosslinked analogues showed poorer cell attachment or greater cytotoxicity [158].

Photocrosslinked gelatin systems extend these concepts toward spatially controlled and injectable biofabrication. Visible-light-crosslinked GelMA hydrogels based on eosin Y chemistry provided tunable mechanical properties and favorable in vitro cardiomyocyte responses; the material was further evaluated through ex vivo myocardial injection and in vivo gelation in infarcted rat hearts [164]. Visible-light-crosslinked GelMA/HAMA hydrogels were also developed to reproduce features of adipose extracellular matrix. In these systems, Ru/SPS concentration controlled swelling and stiffness, while hyaluronic acid provided biochemical cues that supported preadipocyte differentiation [165]. Biofabrication is not limited to photoinduced systems. Three-dimensional bioprinted genipin-crosslinked gelatin hydrogels loaded with hUCMSC-derived small extracellular vesicles combined structural support with paracrine bioactivity and improved dermal fibroblast viability, attachment and migration [166].

Mechanistically, crosslinking defines the cellular microenvironment by jointly regulating matrix stiffness, pore accessibility, degradation, molecular transport, and the availability of cell-interactive motifs. Greater stabilization may support cell adhesion and dimensional integrity, but excessive network densification can restrict spreading, migration, matrix remodeling, and differentiation when transport, degradability, or ligand accessibility become limiting; the optimal network therefore depends on the cell type and target tissue [167].

For regenerative applications, the central design requirement is therefore to provide sufficient mechanical and dimensional stability while preserving the hydration, molecular transport, degradability, and cellular accessibility required for tissue remodeling. The preferred crosslinking route should consequently reflect both the mechanical demands of the target tissue and the need for cytocompatible processing, particularly when cells are encapsulated or gelation occurs in situ.

More broadly, biomedical translation requires evaluation beyond short-term cytocompatibility, including long-term degradation, host immune response, residual crosslinker or reactive species, and batch-to-batch reproducibility under application-relevant conditions.

**Figure 3:**
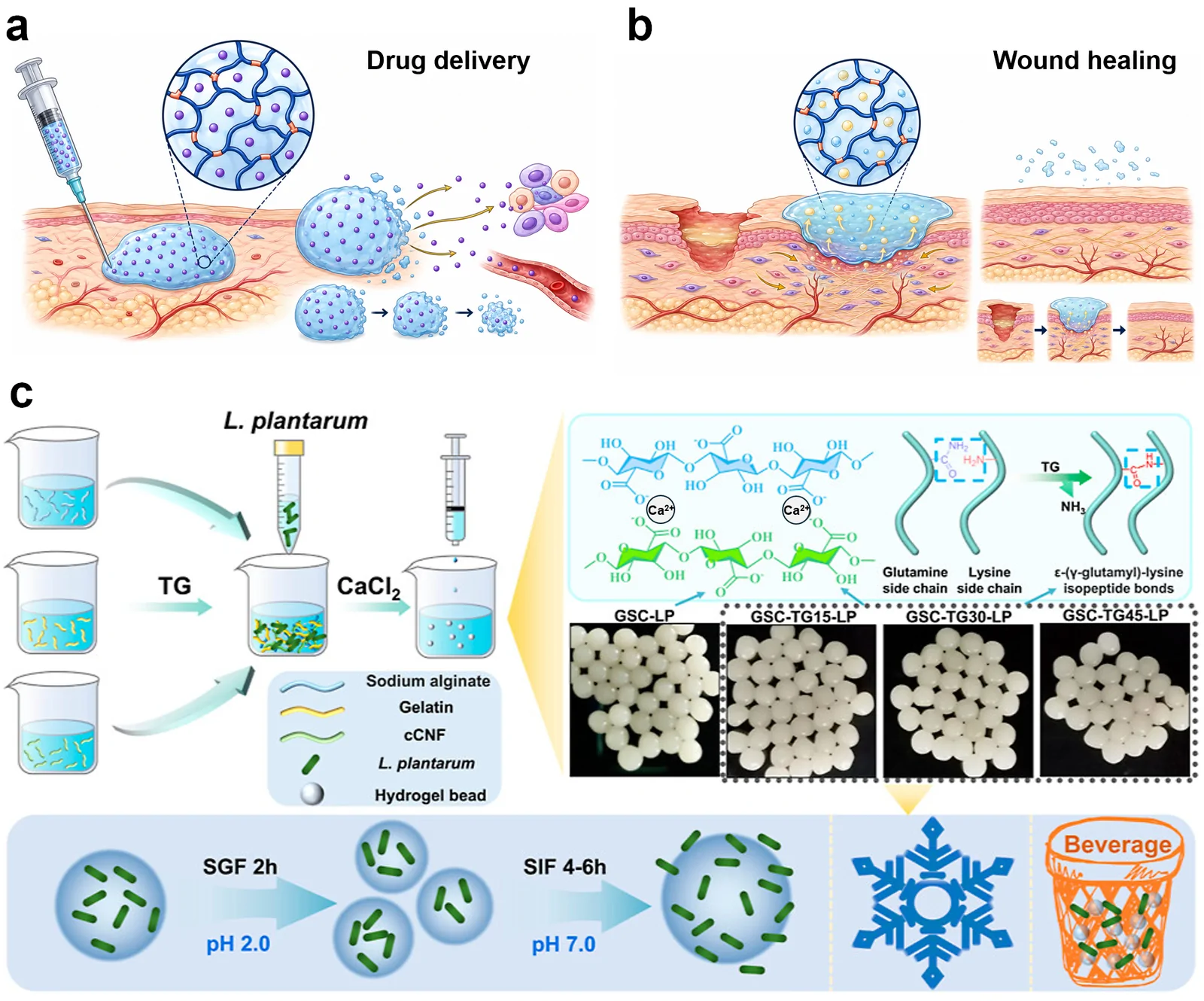
(**a**) Schematic representation of an injectable hydrogel depot for localized drug delivery, illustrating drug encapsulation, controlled release, and progressive matrix degradation. (**b**) Schematic of a gelatin-based wound dressing, showing application to the wound bed, local release of bioactive compounds, cellular infiltration, neovascularization, and tissue regeneration. In (**a**), purple dots represent drug molecules, while in (**b**), yellow dots represent bioactive compounds. (**c**) Preparation and gastrointestinal behavior of dual-crosslinked gelatin–polysaccharide beads for probiotic delivery. Lactiplantibacillus plantarum was incorporated into a gelatin/sodium alginate/carboxylated cellulose nanofiber matrix. Transglutaminase catalyzed the formation of ε-(γ-glutamyl)lysine isopeptide bonds between gelatin chains, whereas Ca^2+^ coordinated the carboxylate-rich polysaccharide phase. Beads prepared without transglutaminase or after 15, 30, and 45 min of enzymatic treatment protected the probiotics in simulated gastric fluid (SGF; pH 2.0, 2 h) and subsequently swelled to enable sustained release in simulated intestinal fluid (SIF; pH 7.0, 4–6 h). Freeze-drying resistance and storage in beverage matrices are also illustrated. Adapted with permission from [169].

For translational biomedical applications, sterilization should also be regarded as a network-processing variable rather than a neutral terminal step. In GelMA and related gelatin hydrogels, autoclaving, ethylene oxide treatment, and γ-irradiation can alter stiffness, swelling, degradation, chain organization, and printability, with the magnitude and direction of these effects depending on formulation and processing stage. The sterilization method should therefore be selected and validated together with the crosslinking strategy, because the properties of the post-sterilization network ultimately determine biomedical performance [168]. Figure 3 provides representative examples of gelatin-based hydrogels for drug delivery, wound healing, and probiotic delivery.

### 4.3. Food Packaging, Preservation and Delivery

Food packaging, preservation, and delivery represent attractive application fields for crosslinked gelatin hydrogels. Gelatin combines film-forming ability, biodegradability, food relevance, and amphiphilic character with the possibility of tuning water uptake, gas transport, mechanical resistance, and release behavior through crosslinking. In these applications, crosslinking not only prevents premature dissolution but also transforms gelatin into an active matrix able to protect packaged products, encapsulate functional ingredients, and regulate their release during storage or digestion.

Enzymatic crosslinking is especially relevant to food systems because microbial transglutaminase (mTG) forms ε-(γ-glutamyl)lysine bonds under mild and food-compatible conditions. In packaging films, mTG has been used to improve gelatin composites such as extruded gelatin–beeswax systems by balancing processability, mechanical integrity, and barrier performance [170]. Enzymatically crosslinked gelatin networks can also protect and deliver probiotic microorganisms. Gelatin/PVA hydrogels crosslinked with mTG were designed to encapsulate Lactiplantibacillus plantarum, preserving probiotic viability after lyophilization and under simulated gastric conditions before release in intestinal fluid [171]. Gelatin/sodium hexametaphosphate hydrogels further showed that enzyme concentration must be carefully optimized. Moderate mTG levels improved texture and network formation, whereas excessive crosslinking restricted gelatin-chain reorganization and produced a less effective three-dimensional structure [172]. Dual-crosslinked gelatin–polysaccharide beads provide a related strategy in which enzymatic and Ca^2+^-mediated junctions enhance probiotic protection and regulate release during gastrointestinal transit (Figure 3c) [169].

Chemical crosslinking provides another route to reinforce gelatin packaging films, although crosslinker selection strongly affects safety, optical properties, and water transport. Low glutaraldehyde concentrations can reduce solubility and water sensitivity, improve water-vapor barrier properties, and increase stiffness. These benefits may be accompanied by reduced extensibility, greater opacity, and concerns related to residual toxicity, which restrict its suitability for edible or direct food-contact materials [173]. Gelatin/CNC hydrogel films illustrate the same trade-off. Glutaraldehyde produced greater mechanical strength, whereas citric acid generated clearer films with lower water permeability and higher swelling capacity. Cellulose nanocrystals further reinforced the matrix and reduced mass loss, although their effect depended on concentration, dispersion, and compatibility with the crosslinked gelatin network [174].

Crosslinker selection in food-related gelatin systems must therefore account not only for network performance but also for safety and suitability for the intended use. Glutaraldehyde, despite its effectiveness in improving water resistance and mechanical stability, raises toxicity concerns that limit its applicability in edible and direct food-contact materials, whereas enzymatic crosslinking with microbial transglutaminase and naturally derived agents such as genipin have been increasingly explored as safer alternatives [175,176,177]. Nevertheless, the suitability of a given crosslinker ultimately depends on its concentration, residual content, migration potential, and the specific food-contact or edible application. Accordingly, successful laboratory-scale performance should not be interpreted as evidence of suitability for food-contact or edible use, which additionally requires compliance with the relevant safety, migration, and residual-content requirements for the intended application.

Renewable crosslinking components have also been investigated for active packaging. A biomass-derived aldehyde combined with aminated graphene oxide enabled multipoint interactions with gelatin through aldehyde–amine reactions and hydrogen bonding. The resulting films exhibited improved tensile strength, thermal stability, hydrophobicity, and water- and gas-barrier performance [178]. Genipin-crosslinked carboxymethyl chitosan/gelatin pads loaded with thyme essential oil provide a complementary humidity-responsive concept. The matrix absorbed moisture and released the antimicrobial oil more rapidly at high relative humidity, matching the conditions that favor microbial spoilage. This behavior preserved blueberry quality and suppressed mold development during storage [179].

Photocrosslinked gelatin derivatives extend this field toward surface-applied coatings for fruit preservation. GelMA films containing chitosan–cinnamaldehyde microspheres combine light-triggered network formation with antibacterial, antioxidant, and UV-shielding functions. Schiff-base formation between chitosan and cinnamaldehyde stabilizes the active microspheres, while the GelMA network provides a flexible coating on fruit surfaces. The resulting films increased water contact angle, reduced UV-C transmittance, enhanced antioxidant capacity, and delayed browning, softening, weight loss, and microbial growth in cherries and bananas [140].

Gelatin hydrogels can also act as internal food-structuring and delivery platforms. mTG-crosslinked fish-gelatin emulsion gels containing β-carotene showed that crosslinking time controls rigidity, thermal irreversibility, deformation resistance, storage stability, and digestion behavior. Intermediate crosslinking times improved gel integrity at room and physiological temperatures, protected β-carotene from degradation, enhanced gastric stability, and increased bioaccessibility. Prolonged crosslinking instead reduced flexibility and energy dissipation, highlighting the need to preserve sufficient molecular mobility within the stabilized network [180].

Accordingly, crosslinking in food-related gelatin systems should improve structural and barrier performance without compromising food compatibility or the molecular mobility required for controlled release, digestion, or active functionality.

### 4.4. Water Remediation and Environmental Management

Gelatin-based networks have also been explored as bio-based platforms for environmental remediation, particularly for contaminant removal, degradation, and monitoring in water. Their amino, carboxyl, hydroxyl, and amide groups can interact with metal ions and dyes, while the hydrated network can host enzymes, conductive additives, and inorganic particles for catalytic or sensing functions. Crosslinking is essential to prevent dissolution and preserve structural integrity during aqueous operation. Glutaraldehyde-crosslinked gelatin hydrogels reinforced with eucalyptus-derived cellulose residues illustrate this concept. The incorporation of pristine or treated fibers reduced swelling and increased rigidity without suppressing Cr(VI) adsorption, which remained around 12–13 mg g^−1^ and was promoted by hydroxyl-rich surfaces and protonated groups able to interact with chromate species [181].

A more multifunctional approach was reported for gelatin/TiO_2_/PEI aerogels. Gelatin formed the porous scaffold, PEI increased the density of amine-rich binding sites, and TiO_2_ introduced photocatalytic activity. These aerogels combined dye adsorption, Cu(II) removal, oil–water separation, and partial photocatalytic dye degradation, showing how gelatin-based architectures can address wastewater streams containing different pollutant classes [182]. Heavy-metal adsorption has also been investigated using UV-cured methacrylated gelatin and chitosan hydrogels for As(V) and Pb(II) removal. The methacrylated gelatin networks showed swelling degrees of up to approximately 1200%, while adsorption depended strongly on pH, surface charge, network swelling, and ion speciation. Contaminant capture was therefore governed not only by the chemical identity of the binding groups but also by their accessibility and ionization state within the hydrated network [183].

Glutaraldehyde-crosslinked gelatin/chitosan hydrogels have also been evaluated for dye removal. These materials adsorbed acid fuchsin, methyl orange, crystal violet, Congo red, malachite green, and methylene blue. Their adsorption behavior followed Langmuir-type isotherms and pseudo-second-order kinetics, while removal efficiencies remained above 50% after five adsorption–desorption cycles, indicating partial recyclability [184].

Beyond dye removal, gelatin/chitosan networks have been investigated for selective metal-ion capture. Spherical chitosan/gelatin hydrogel particles prepared by inverse emulsion showed high selectivity for Hg(II) in single-metal solutions, particularly at higher chitosan contents, due to favorable coordination with nitrogen-containing groups. In mixed solutions containing Pb(II), Cd(II), Hg(II), and Cr(III), removal efficiencies reached approximately 73–94%, showing that multicomponent conditions can substantially modify adsorption behavior relative to single-ion systems [185]. Functionalized magnetic architectures provide an additional strategy for sorbent recovery. Chitosan/PEI-grafted magnetic gelatin crosslinked with transglutaminase retained amino groups for subsequent functionalization and metal binding, while its magnetic character enabled rapid separation. The resulting sorbents showed high adsorption capacities for Pb(II) and Cd(II), together with good stability, regeneration, and effective removal at concentrations relevant to drinking-water treatment [186].

Gelatin matrices can also support active contaminant degradation and environmental monitoring rather than serving only as passive adsorbents. Glutaraldehyde-crosslinked gelatin beads were used for laccase immobilization, producing reusable biocatalysts for water-treatment-related oxidation reactions. The immobilized enzyme retained approximately 60% of its initial activity after ten cycles and showed greater thermal stability than free laccase [187]. GelMA-based electroactive living materials containing Ca^2+^-doped PEDOT:PSS and Shewanella oneidensis further extended gelatin platforms toward environmental sensing. These 3D-bioprinted biosensors responded rapidly to Cr(VI), nitrobenzene, and chloroform and maintained improved operational stability in real-water samples [188].

From a practical perspective, the performance metrics reported for remediation systems require careful interpretation. Adsorption capacities and removal efficiencies should be considered together with the experimental conditions under which they were obtained, including initial pollutant concentration, sorbent dose, pH, competing ions, contact or equilibrium time, regeneration protocol, and the use of model solutions versus real wastewater. Values obtained under substantially different conditions are therefore not directly comparable. For prolonged aqueous operation, practical evaluation should also consider network mass and dimensional stability, gelatin dissolution or degradation, and possible leaching of incorporated polymers, particles, catalysts, or other secondary components during repeated use.

Gelatin should not, however, be regarded as intrinsically superior to other biopolymer or synthetic hydrogel matrices for water remediation. Its main value lies in combining a chemically functional and biodegradable protein scaffold with straightforward aqueous processing and compatibility with secondary sorbents, catalysts, and functional polymers; in many high-performance systems, selectivity and adsorption capacity are nevertheless largely determined by components such as chitosan, PEI, TiO_2_, or other incorporated functionalities rather than by gelatin alone [182,189,190,191,192].

Across environmental applications, crosslinking should provide sufficient aqueous stability and recyclability while preserving the accessibility of adsorption, catalytic, or sensing sites within the hydrated network. Network stabilization is therefore most effective when it supports functional-group accessibility and mass transport rather than simply maximizing rigidity or crosslink density.

### 4.5. Wearable Sensing and Bioelectronics

**Figure 4:**
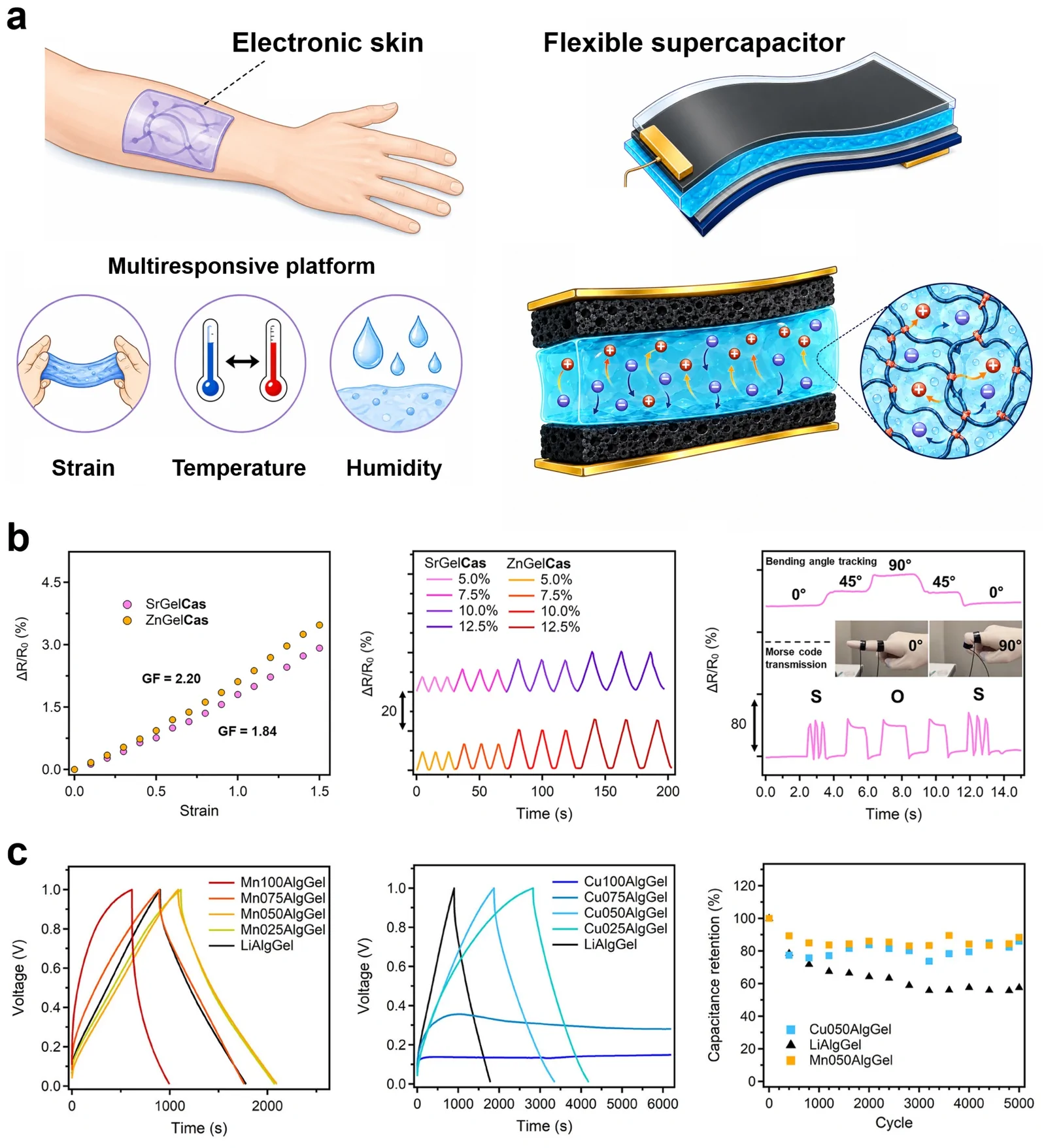
(**a**) Illustration of hydrogel-based soft electronics, including electronic skin, flexible supercapacitors, and multiresponsive sensing of strain, temperature, and humidity. The device cross-section highlights ion transport through the hydrogel electrolyte. (**b**) Piezoresistive performance of Sr^2+^- and Zn^2+^-crosslinked caseinate–gelatin–glycerol organohydrogels (SrGelCas and ZnGelCas). Relative resistance changes (ΔR/R_0_) under tensile deformation show linear responses with gauge factors of 1.84 and 2.20, respectively. Cyclic tests at strain amplitudes of 5.0–12.5% demonstrate reproducible strain-dependent signals, while SrGelCas enables real-time bending-angle detection from 0° to 90° and transmission of the Morse-code sequence “SOS”. Adapted with permission from [38]. (**c**) Electrochemical performance of Mn^2+^- and Cu^2+^-crosslinked gelatin–alginate organohydrogel electrolytes containing different Li^+^ proportions. Galvanostatic charge–discharge curves of the MnXAlgGel and CuXAlgGel series and LiAlgGel were recorded at 0.2 mA cm^−2^. Capacitance retention of Mn050AlgGel, Cu050AlgGel, and LiAlgGel over 5000 charge–discharge cycles is also shown. Adapted with permission from [121].

Wearable sensing is one of the most active directions for crosslinked gelatin hydrogels because it requires materials that combine softness, deformability, conductivity and stability under repeated deformation and prolonged contact with the body (Figure 4a). Gelatin provides a hydrated and tissue-like matrix whose mechanical and electrical properties can be tuned through covalent crosslinking, salt-induced association, conductive fillers and solvent engineering. Genipin-crosslinked gelatin hydrogels reinforced through the Hofmeister effect showed high tensile and compressive resistance, stretchability above 500%, freezing tolerance and strain sensitivity suitable for monitoring human motion [193]. Fish-gelatin/gellan-gum biogels processed by transglutaminase crosslinking and ethanol treatment enabled 3D-printed biodegradable strain sensors with rapid response, high sensitivity and durability under repeated deformation [194]. Patterned AgNW–GelMA systems further showed how gelatin-derived networks can stabilize conductive pathways, with chemical welding improving nanowire junctions and electrical response to small mechanical stimuli [195]. Gelatin hydrogels containing AgNWs/PEDOT:PSS and stabilized by glutaraldehyde crosslinking were also developed as low-hysteresis sensors for monitoring handwriting, arterial pulse and facial expressions, with wireless readout and machine-learning-assisted motion classification [196].

Gelatin-based organohydrogels and ionically conductive hybrid networks provide another route to durable sensing. Glycerol, salts and charged biopolymers improve water retention, flexibility and ionic transport under changing environmental conditions. Ion-crosslinked alginate/gelatin and caseinate/gelatin (Figure 4b) organohydrogels showed that the identity of the coordinating metal ion can regulate network mechanics, conductivity and multifunctional response, enabling strain, temperature, humidity and light detection in transparent stretchable matrices [37,38]. Fully bio-based gelatin/glycerol organohydrogels stabilized by transglutaminase demonstrated that electronic fillers are not always required. In these materials, mobile ions within the hydrated glycerol-rich network provided conductivity, while enzymatic crosslinking preserved mechanical integrity and enabled large-strain sensing, thermal response and long-term cycling stability [197]. Other enzymatic systems incorporated Z–Gln–Gly into gelatin, combining transglutaminase-mediated covalent coupling with dynamic π–π interactions to improve toughness, conductivity and pressure sensitivity [198]. Conductive fillers nevertheless remain useful when higher or more stable electronic transport is required, as shown by gelatin hydrogels containing sandwich-like PPy/rGO/PPy nanosheets for simultaneous strain, pressure and temperature sensing [199].

Gelatin hydrogels are also being integrated into broader bioelectronic formats. Genipin-crosslinked gelatin films patterned with liquid-metal/PVP conductors were developed as flexible patches for cardiac and neural signal acquisition, combining wet adhesion, biocompatibility, flexural fatigue resistance and stable in vivo ECG and EEG monitoring [200]. Gelatin methacryloyl granular scaffolds have been incorporated into microfluidic wearable devices for collecting ultralow sweat volumes at rest. Coupling these scaffolds with laser-induced graphene electrodes and pH correction enabled continuous lactate sensing [201]. These systems extend gelatin from a compliant sensing matrix to an interface for biofluid collection, electrophysiological recording and physiological monitoring.

From a practical perspective, short-term sensitivity or gauge factor alone is insufficient to establish wearable performance. Evaluation should also consider signal hysteresis and drift over repeated deformation, mechanical fatigue, dehydration and water retention, adhesion during prolonged skin contact, and stability in sweat or other relevant biofluids; where required for the intended use, the effects of sterilization on network and sensing performance should also be verified.

Overall, crosslinking in wearable gelatin hydrogels should provide sufficient mechanical stabilization to minimize hysteresis, drift, and structural fatigue without excessively restricting the molecular or ionic mobility responsible for transduction. Ionic and organohydrogel networks are particularly attractive when conductivity and water retention can be maintained without rigid conductive fillers, whereas electronically conductive components become advantageous when higher or more stable charge transport is required.

### 4.6. Energy Storage

Gelatin-based hydrogels have recently emerged as attractive electrolyte matrices for soft energy-storage devices (Figure 4c), particularly flexible supercapacitors and quasi-solid-state Zn batteries. In these systems, gelatin is rarely used as a passive scaffold. Its hydrated network, polar functional groups and processability are instead exploited to combine ion transport, mechanical compliance, electrolyte retention and stable electrode contact. Dense covalent crosslinking is often avoided in supercapacitor electrolytes because excessive network fixation may restrict ionic mobility and reduce interfacial adaptability. Many formulations therefore rely on physical, ionic or hybrid interactions to preserve both conductivity and deformability.

In early flexible-supercapacitor designs, gelatin hydrogels were mainly engineered as quasi-solid-state electrolytes through physical reinforcement and salt-mediated structuring. Gelatin/cellulose-nanofiber hydrogels used Hofmeister-type salting-out effects and nanofiber reinforcement to improve mechanical stability while maintaining ion transport, enabling flexible carbon-cloth supercapacitors with stable capacitive behavior and satisfactory cycling retention [202]. Similar principles were applied to gelatin/chitosan electrolytes treated with ammonium sulfate and ammonium chloride. Sulfate-induced salting-out and chitosan precipitation reinforced the network, while the mixed-salt environment preserved ionic conductivity and low-temperature operation, with conductivities in the order of tens of mS cm^−1^ [203]. Hybrid PVA/gelatin/LiCl matrices further illustrate this design philosophy. Gelatin contributes polar groups and water retention, while the PVA-rich network and LiCl improve mechanical integrity, frost resistance and ion transport, allowing the same hydrogel to function as both a strain-sensitive material and a supercapacitor electrolyte [204].

More recent studies have moved beyond simple electrolyte replacement toward integrated and multifunctional supercapacitor architectures. Fe^3+^-doped gelatin/poly(acrylate-co-acrylamide) hydrogels stabilized through hydrogen bonding, metal coordination and glycerol-assisted water retention have been used for biopotential acquisition, salt-solution recognition and flexible supercapacitors [205]. These systems illustrate how sensing and energy storage can be combined within a single hydrated platform. Structural control of the electrolyte provides another important strategy. Directionally frozen gelatin/poly(methacrylic acid) hydrogels containing oriented porous channels promoted ion transport and enabled polypyrrole growth within the hydrogel framework, improving electrode–electrolyte contact in all-in-one supercapacitors. After glycerol incorporation, the devices retained 77.2% of their capacitance at −23 °C and remained stable under stretching, compression and bending, demonstrating how pore orientation and solvent engineering can reduce interfacial resistance and suppress freezing [206].

Ionically crosslinked gelatin-based organohydrogels provide a particularly clear example of rational electrolyte design. In gelatin–alginate systems, Cu^2+^ or Mn^2+^ crosslinking combined with Li^+^ incorporation allowed the nanoscale network structure to be tuned without covalent modification. Cu^2+^ produced compact and mechanically stable networks, whereas Mn^2+^ generated looser structures that became more flexible after Li^+^ incorporation. This balance strongly influenced device performance: Cu^2+^-crosslinked gels achieved high areal capacitance and energy density, while Mn^2+^-crosslinked gels showed superior capacitance retention over 5000 cycles [121]. These findings show that crosslinking is most effective in supercapacitor electrolytes when it simultaneously regulates ion-transport pathways, solvent retention and mechanical integrity.

Gelatin hydrogels have also been extended to more specialized energy-storage concepts. In ionic thermoelectric supercapacitors, gelatin–salt hydrogels act not only as charge-storage electrolytes but also as media for thermally driven ion redistribution. Electrode–electrolyte interactions can substantially alter the measured thermopower and even switch the apparent response from p-type to n-type behavior [207]. Covalent crosslinking becomes more relevant when the electrolyte must form a free-standing membrane or regulate metal deposition. Glyoxal- and glutaraldehyde-crosslinked gelatin membranes containing hydroquinone have been investigated as free-standing redox-active electrolytes for solid-state electric double-layer capacitors, although their conductivity and capacitance remain moderate compared with more ion-rich organohydrogels [208].

Chemically reinforced or dual-crosslinked gelatin electrolytes are particularly attractive for Zn metal batteries because they must suppress dendritic growth, reduce water activity, regulate Zn^2+^ desolvation and stabilize the electrode interface. Gelatin/oxidized-dextran/methacrylic-anhydride networks enabled prolonged Zn plating–stripping and supported 10,000-cycle operation in Zn||I_2_ full cells [209]. Dual-crosslinked gelatin/PAM hydrogels improved Zn^2+^ coordination and interfacial compatibility, extending Zn||Zn cycling beyond 1500 h [210]. The preferred crosslinking strategy is therefore strongly device-dependent: physical and ionic networks are frequently favored in flexible supercapacitors, whereas chemically reinforced networks become advantageous when dimensional stability and metal-anode regulation are decisive.

Gelatin is likewise not intrinsically a higher-performance electrolyte matrix than other biopolymer or synthetic hydrogels. Its particular interest arises from the combination of a hydrated polar network, facile crosslinking, mechanical compliance, and compatibility with salts, secondary polymers, and solvent engineering, while the final conductivity and electrochemical performance often depend strongly on these additional components rather than on gelatin alone.

At the device level, ionic conductivity should not be treated as an isolated measure of electrolyte performance. Practical evaluation should also consider the electrochemical stability window, electrode–electrolyte compatibility, solvent and water retention, temperature-dependent transport and mechanical behavior, and retention of electrochemical and interfacial performance during prolonged cycling.

Across these systems, optimal electrolyte design therefore requires balancing ionic mobility and solvent retention against mechanical stability and interfacial control rather than maximizing crosslink density. Table 3 provides a comparative overview of the application-specific design requirements discussed in Section 4.

## 5. Conclusions and Outlook

Crosslinking has transformed gelatin from a thermoreversible protein gel into a versatile platform for functional soft materials. The evidence examined in this review shows that hydrogel performance is governed not simply by the number of crosslinks but by the chemistry, distribution, dynamics and hierarchical organization of the network junctions. Crosslinking therefore acts as a molecular design tool that controls hydration, mechanics, molecular and ionic transport, degradation and responsiveness to external stimuli.

The available strategies occupy distinct but complementary design spaces. Temperature-induced assembly preserves the intrinsic thermoreversible character of gelatin but provides limited environmental stability, while ion-mediated interactions introduce dynamic and responsive reinforcement. Covalent crosslinkers, coupling agents and enzymatic reactions provide more persistent stabilization, whereas photocrosslinking enables spatially and temporally controlled network formation. Combining permanent and reversible junctions within hybrid networks is particularly promising because these interactions can provide complementary functions, including structural integrity, energy dissipation, adhesion, conductivity and recovery after deformation.

These differences become decisive when gelatin hydrogels are designed for drug delivery and controlled release, tissue engineering and wound healing, food packaging, preservation and delivery, water remediation and environmental management, wearable sensing and bioelectronics or energy storage. Across these fields, the central challenge is not to maximize crosslinking but to balance network stability with the molecular mobility required for a specific function. Dense networks may improve stiffness and resistance to dissolution while restricting swelling, diffusion, degradation, ionic transport or large deformation. More dynamic networks instead favor transport and responsiveness but may suffer from mechanical drift, dehydration or limited durability. Meaningful structure–property–function relationships must therefore be established by integrating chemical, structural, thermal, mechanical and functional measurements under conditions representative of the intended application.

Future progress will depend on translating these relationships into predictive design rules. Machine-learning approaches may provide a practical route toward this objective. Artificial neural networks have already been applied to gelatin-based hydrogels to predict formulation-dependent mechanical and gelation behavior, while more recent models have been used to estimate rheological properties and guide hydrogel composition selection [211,212]. Extending these approaches to gelatin hydrogel design will nevertheless require sufficiently large and standardized datasets accounting for gelatin source, molecular-weight distribution, Bloom strength, concentration, functionalization, crosslinking conditions, hydration state, thermal history, and testing protocols. Greater control over gelatin source, molecular-weight distribution, Bloom strength, functionalization, hydration and thermal history is required to improve reproducibility and enable reliable comparisons among studies. Hybrid networks, safer bio-derived crosslinkers, visible-light reactions and scalable aqueous processing offer promising routes toward more sustainable and durable materials. Standardization efforts already exist for specific hydrogel applications; for example, ASTM F2900-25 provides guidance for the characterization of hydrogels used in regenerative medicine, including network formation, degradation, agent release, stability, and mass transport [213]. Broader harmonization of testing under realistic mechanical and environmental conditions will nevertheless be important for enabling reliable comparisons and distinguishing short-term demonstrations from technologies capable of sustained operation. The future of gelatin hydrogels lies not in producing stronger gels alone but in programming the appropriate balance of stability, mobility, degradation and responsiveness for a defined function.

## Figures and Tables

**Table 1:** Comparison of the principal non-covalent and covalent strategies used to form and stabilize gelatin hydrogel networks, including the gelatin sites or structural motifs involved, junction type, reversibility, principal advantages, and main limitations.

Network Formation/Stabilization Strategy	Gelatin Sites Involved	Junction Formed	Reversibility *	Principal Advantages	Main Limitation
Thermal physical gelation [12,29,30,31]	Collagen-like sequences	Triple-helical junction zones	Reversible	Mild and reagent-free	Limited thermal and aqueous stability
Ion-mediated association/reinforcement in gelatin [14,34,35,36]	Charged and coordinating residues	Screening/coordination-assisted associations	Dynamic	Simple and stimuli- responsive	Ion- and condition-dependent
Ionic crosslinking in gelatin-containing hybrid networks [37,38,39,40,41]	Mainly ionic groups of the secondary polymer	Multivalent-ion- mediated junctions	Dynamic	Rapid aqueous gelation and tunability	Junctions may primarily involve the secondary polymer
Glutaraldehyde crosslinking [44,45,46,47]	Primary amino groups	Complex aldehyde- derived covalent junctions	Irreversible	Rapid and efficient stabilization	Cytotoxicity and residual reactivity
Oxidized polysaccharide crosslinking [48,49,50]	Primary amino groups	Dynamic imine bonds	Partially reversible	Biopolymer-based, self-healing potential	Hydrolytic instability
Genipin crosslinking [16,27,51,52,53,54]	Primary amino groups	Amine-derived covalent junctions	Irreversible	Lower cytotoxicity than glutaraldehyde	Slow reaction
EDC/NHS coupling [55,56,57,58,59]	Amino and carboxyl groups	Direct amide bonds	Irreversible	Zero-length coupling	Sensitive to pH and stoichiometry
Enzymatic crosslinking [42,60,61,62,63,64,65,66,67,68]	Native residues or phenolic/catechol groups	Isopeptide bonds or oxidative covalent junctions	Irreversible	Selective reactions under mild aqueous conditions	Cost and kinetic limitations
Photocrosslinking [28,43,69,70,71,72,73,74,75,76,77,78,79,80,81]	Methacryloyl, norbornene, or native tyrosine residues	Chain-growth, thiol–ene, or dityrosine covalent networks	Irreversible	High spatial and temporal control	Functionalization and/or photochemical requirements

* Reversibility refers to the physicochemical reversibility of the network junctions under non-degradative conditions and does not imply resistance to enzymatic or hydrolytic degradation of the gelatin network. Thermal gelation forms reversible physical junctions, whereas ion-mediated associations and ionic hybrid crosslinking are dynamic non-covalent. Oxidized-polysaccharide systems involve partially reversible dynamic imine bonds, while glutaraldehyde, genipin, EDC/NHS, enzymatic, and most photocrosslinking routes form persistent covalent junctions that are essentially irreversible under non-degradative conditions. These descriptors refer to the dominant junction type, although multiple junctions may coexist within the same gelatin network.

**Table 2:** Representative quantitative effects of crosslinking strategy and processing variables on the physicochemical properties of gelatin-based hydrogels.

Crosslinking Route/Variable	Representative System	Quantitative Effect	Key Implication	Ref.
Hofmeister-mediated physical reinforcement	Gelatin hydrogels soaked in Na_2_SO_4_ solutions	Gelation temperature broadened from 32 to 46 °C; maximum stress increased ~40-fold to 0.835 MPa and strain ~7-fold to 238%	Strong ion-dependent reinforcement	[144]
Genipin–crosslinking temperature	Gelatin crosslinked with genipin at 5, 15, and 25 °C	Crosslinking index increased from 20.5% → 76.5% → 88.0%	Strong processing dependence	[145]
Crosslinker chemistry	Same gelatin-sponge system crosslinked with GTA, genipin, EDC, or mTG	Compressive modulus (hydrated state): 154/261/56.6/67.4 kPa; swelling: 956/940/1388/1209%; mass remaining after 5 months: ~94/~98/54.3/~94%, respectively	Chemistry controls mechanics and persistence	[146]
mTG–gelatin type and enzyme dose	Type A and type B gelatin (3 wt%), 0–20 U mTG g^−1^ gelatin	Type A gelatin lost thermoreversibility at ≥12 U g^−1^, whereas type B retained a sol–gel transition throughout the investigated 0–20 U g^−1^ range	Gelatin source governs response	[61]
GelMA–degree of methacryloylation	GelMA with 49.8, 63.8, and 73.2% functionalization	Compressive modulus: 2.0 → 3.2 → 4.5 kPa; pore size: 49.7 → 30.1 → 23.6 μm; complete collagenase degradation extended from 6 to 15 h between the lowest and highest substitution	Higher substitution increases stability	[147]
GelMA–physical + covalent crosslinking	4–6 wt% porcine, bovine, or cold-water-fish GelMA photocrosslinked after different thermal histories	G′ ranges: porcine 723–7340 Pa, bovine 516–3484 Pa, fish 294–464 Pa; physical gelation before photocuring produced up to ~8-fold higher G′ than chemical crosslinking alone	Thermal history strongly affects stiffness	[43]
Ru/SPS dityrosine photocrosslinking	Unmodified 10% gelatin, Ru/SPS 1/10 mM, visible light 30 mW cm^−2^ for 1 min after physical gelation	Photocrosslinking stabilized pristine gelatin at 37 °C, whereas the native physical gel dissolves above ~25 °C	Stabilization without derivatization	[78]

Values are intended to illustrate representative magnitudes and within-study trends rather than establish universal rankings among crosslinking strategies. Direct comparison of absolute values across studies is limited by differences in gelatin source and concentration, crosslinking conditions, hydration state, specimen geometry, and testing protocols. Accordingly, a minimum reporting set for reproducible cross-study comparison should include, where applicable, gelatin source and type (A/B), Bloom strength and/or molecular-weight distribution, gelatin concentration, pH and ionic strength, thermal history, degree of functionalization, crosslinker identity, purity and concentration, reaction time and temperature, purification procedure, hydration state, and relevant testing conditions.

**Table 3:** Application-specific network requirements, crosslinking objectives, key performance indicators and principal design trade-offs of gelatin hydrogels.

Application	Required Network Characteristics	Crosslinking Design Objective	Key Performance Indicators	Principal Trade-Off	Ref.
Drug delivery and controlled release	Controlled swelling and degradation	Tune drug retention and release	Encapsulation efficiency, burst release, release kinetics, degradation	Retention versus biodegradability	[91,145,148,149,150,151,152,153,154,155,156,157]
Tissue engineering and wound healing	Cytocompatibility, porosity and suitable stiffness	Support integrity and cell remodeling	Cell viability, migration, modulus, degradation, tissue formation	Stability versus biological accessibility	[63,158,159,160,161,162,163,164,165,166,167,168,169]
Food packaging, preservation and delivery	Moisture control, barrier performance and safety	Improve stability and active release	Water-vapor permeability, tensile properties, solubility, release, shelf life	Barrier performance versus safety	[140,169,170,171,172,173,174,175,176,177,178,179,180]
Water remediation and environmental management	Accessible binding sites and water stability	Preserve adsorption and regenerability	Adsorption performance, selectivity and regeneration	Stability versus site accessibility	[181,182,183,184,185,186,187,188]
Wearable sensing and bioelectronics	Elasticity, conductivity and water retention	Stabilize signals during deformation	Gauge factor, response time, cycling stability	Durability versus sensitivity	[37,38,121,193,194,195,196,197,198,199,200,201]
Energy storage	Ionic conductivity, solvent retention and electrode contact	Balance stability and ion transport	Conductivity, capacitance and cycling stability	Stability versus ion mobility	[121,202,203,204,205,206,207,208,209,210]

Representative crosslinking strategies discussed for each application include covalent, enzymatic, and photo-crosslinking for drug delivery and tissue engineering; enzymatic and selected covalent or hybrid approaches for food-related systems; covalent or enzymatic stabilization, often within hybrid networks, for water remediation; ionic/physical, enzymatic, and hybrid strategies for wearable sensing; and ionic/physical or covalent/dual-crosslinked networks for energy storage, depending on the device architecture. These associations are representative rather than prescriptive, as the optimal strategy depends on formulation and operating conditions.

## Data Availability

No new data were created or analyzed in this study. Data sharing is not applicable to this article.

## Abbreviations

The following abbreviations are used in this manuscript:

AFM	Atomic force microscopy
AgNW	Silver nanowire
CNC	Cellulose nanocrystal
COL1	Type I collagen
DFO	Deferoxamine
DSC	Differential scanning calorimetry
ECG	Electrocardiography
EDC	1-Ethyl-3-(3-dimethylaminopropyl)carbodiimide
EEG	Electroencephalography
FTIR	Fourier-transform infrared spectroscopy
GelMA	Gelatin methacryloyl
GTA	Glutaraldehyde
HAMA	Methacrylated hyaluronic acid
HEK293	Human embryonic kidney 293 cells
HIF-1α	Hypoxia-inducible factor 1-alpha
HRP	Horseradish peroxidase
hUCMSC	Human umbilical cord mesenchymal stem cell
LAP	Lithium phenyl-2,4,6-trimethylbenzoylphosphinate
mTG	Microbial transglutaminase
NHS	N-Hydroxysuccinimide
NMR	Nuclear magnetic resonance
PAM	Polyacrylamide
PEDOT:PSS	Poly(3,4-ethylenedioxythiophene):poly(styrene sulfonate)
PEI	Polyethylenimine
PPy	Polypyrrole
PVA	Poly(vinyl alcohol)
PVP	Polyvinylpyrrolidone
rGO	Reduced graphene oxide
Ru/SPS	Tris(2,2′-bipyridyl)ruthenium(II)/sodium persulfate photoinitiating system
SANS	Small-angle neutron scattering
SAXS	Small-angle X-ray scattering
SEM	Scanning electron microscopy
SGF	Simulated gastric fluid
SIF	Simulated intestinal fluid
TGA	Thermogravimetric analysis
TNBS	2,4,6-Trinitrobenzenesulfonic acid
UV	Ultraviolet
VEGF	Vascular endothelial growth factor
XPS	X-ray photoelectron spectroscopy
XRD	X-ray diffraction
